# Hyaluronic acid metabolism and chemotherapy resistance: recent advances and therapeutic potential

**DOI:** 10.1002/1878-0261.13551

**Published:** 2024-03-21

**Authors:** Zhanhong Liu, Pengbo Hou, Jiankai Fang, Changshun Shao, Yufang Shi, Gerry Melino, Angelo Peschiaroli

**Affiliations:** ^1^ Department of Experimental Medicine University of Rome Tor Vergata Rome Italy; ^2^ Institutes for Translational Medicine, State Key Laboratory of Radiation Medicine and Protection The First Affiliated Hospital of Soochow University, Suzhou Medical College of Soochow University China; ^3^ Institute of Translational Pharmacology (IFT), National Research Council (CNR) Rome Italy

**Keywords:** cluster of differentiation 44, chemoresistance, extracellular matrix, hyaluronic acid

## Abstract

Hyaluronic acid (HA) is a major component of the extracellular matrix, providing essential mechanical scaffolding for cells and, at the same time, mediating essential biochemical signals required for tissue homeostasis. Many solid tumors are characterized by dysregulated HA metabolism, resulting in increased HA levels in cancer tissues. HA interacts with several cell surface receptors, such as cluster of differentiation 44 and receptor for hyaluronan‐mediated motility, thus co‐regulating important signaling pathways in cancer development and progression. In this review, we describe the enzymes controlling HA metabolism and its intracellular effectors emphasizing their impact on cancer chemotherapy resistance. We will also explore the current and future prospects of HA‐based therapy, highlighting the opportunities and challenges in the field.

## Graphical abstract

Hyaluronic acid (HA), a major component of the extracellular matrix, mediates essential biochemical signals required for tissue homeostasis. Many solid tumors are characterized by dysregulated HA metabolism. Here, we review the current knowledge on the role of HA in cancer chemotherapy and examine the status and prospects of HA‐based cancer therapy, highlighting the opportunities and challenges in this field.

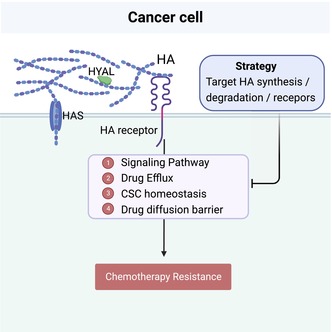

**Abbreviations**
4‐MU: 4‐methylumbelliferone
CD44: cluster of differentiation 44
CSCs: cancer stem cells
ECM: extracellular matrix
EGF‐R: epidermal growth factor receptor
ERK1: extracellular response kinase 1
ERK2: extracellular response kinase 2
FOLFIRI: folinic acid, 5‐FU and irinotecan
GlcNAc: *N*‐acetyl‐d‐glucosamine
GlcUA: d‐glucuronic acid
GSH: glutathione
HA: hyaluronic acid
HAases: hyaluronidases
HARE: HA receptor for endocytosis
HAS: hyaluronan synthases
HMW‐HA: high‐molecular weight HA
HNSCC: head and neck squamous cell carcinoma
IFP: interstitial fluid pressure
LMW‐HA: low‐molecular weight HA
LYVE‐1: lymphatic vessel endothelial hyaluronan receptor 1
MAPK: mitogen‐activated protein kinase
MMPs: matrix metalloproteinases
NFE2L2: nuclear factor erythroid 2‐like 2
PAG: nab‐paclitaxel/gemcitabine
PDA: pancreatic ductal adenocarcinoma
PEGPH20: PEGylated human recombinant PH20 hyaluronidase
PFS: progression‐free survival
PKC: protein kinase C
PKM2: pyruvate kinase M2
RAR: retinoic acid receptor
RHAMM: receptor for hyaluronan‐mediated motility
RNS: reactive nitrogen species
ROS: reactive oxygen species
RTKs: receptor tyrosine kinases
TGFβ: transforming growth factor‐β
TLR‐2: toll‐like receptor 2
TLR‐4: toll‐like receptor 4
TME: tumor microenvironment
UGT: UDP‐glucuronosyltransferase

## 1 Introduction

While chemotherapy is a common and effective treatment option for cancer patients, contributing to kill cancer cells and preventing them from spreading, the response to chemotherapy is often transient. The development of chemoresistance is, indeed, a major challenge in cancer treatment and multiple molecular mechanisms underlie the tumor response to chemotherapy, including the circuits controlling the homeostasis of the tumor microenvironment (TME) [1, 2, 3, 4, 5, 6]. In solid tumors, TME consists of an intricate network of tumor cells, stromal cells, immune cells, and endothelial cells embedded in the extracellular matrix (ECM). The interactions between these components contribute to the development of a complex network that supports tumor growth, invasion, and metastasis. Moreover, TME can also cause drug resistance through various mechanisms, including altered drug metabolism, reduced drug uptake, and activation of signaling pathways that promote cell survival and proliferation [7, 8, 9].

Changes in the composition and structure of the ECM in the TME contribute to chemotherapy resistance in cancer cells. The main components of ECM include collagen, noncollagenous glycoproteins, glycosaminoglycans, and proteoglycans. Hyaluronic acid (HA) is one of the most abundant glycosaminoglycans present in the ECM, playing important physiological functions in many biological processes such as embryonic morphogenesis, cellular regeneration, and wound healing [10]. In addition to providing structural and mechanical support and protection to resident cells, HA impacts a variety of cellular processes, including cell growth, cell survival, migration, invasion, cell fate determination, and tissue morphogenesis.

**Fig. 1:**
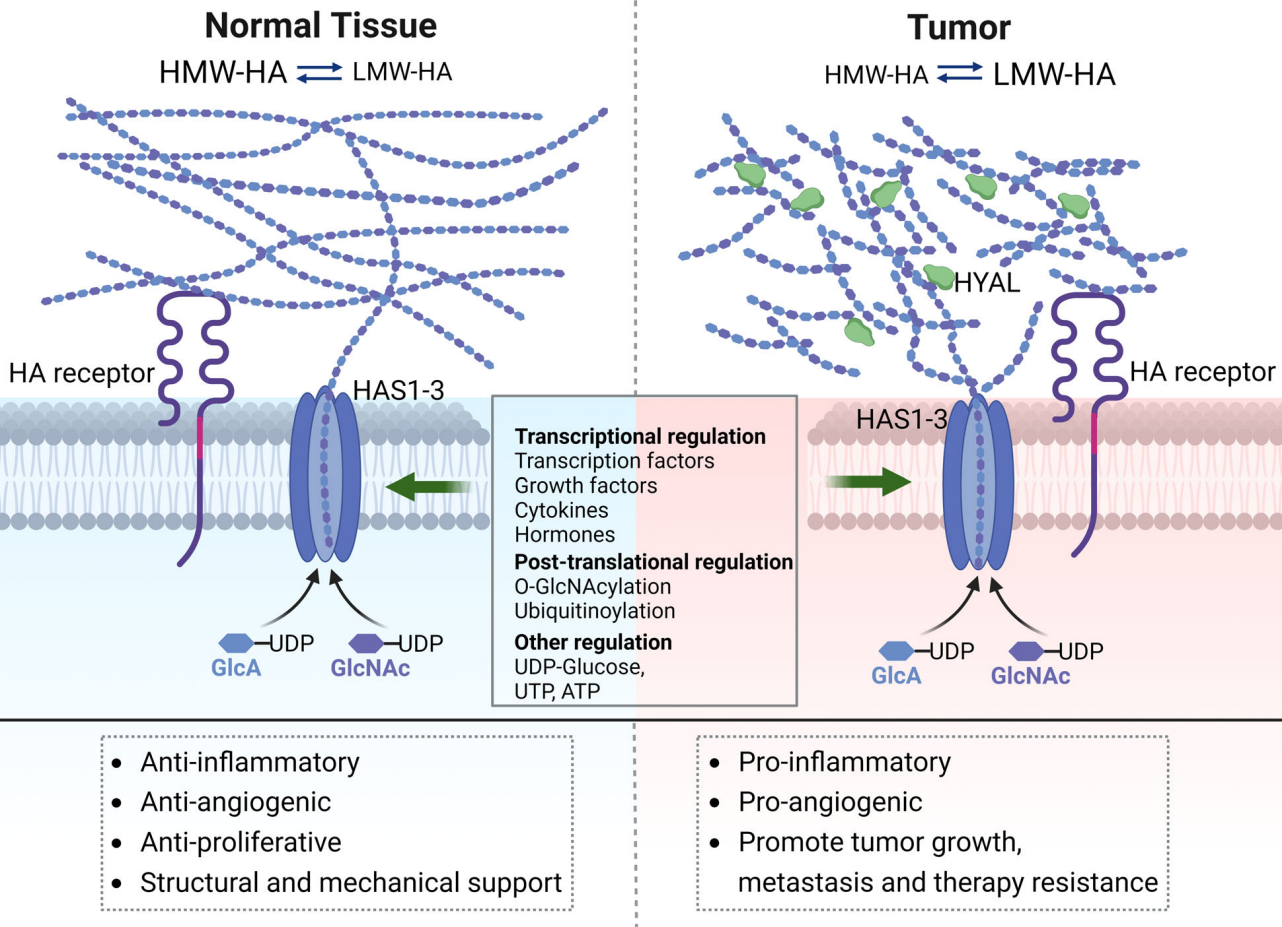
Hyaluronic acid (HA) synthesis, catabolism, regulation, and functions. HA is synthesized at the plasma membrane by the transmembrane hyaluronan synthases (HAS): HAS1, HAS2, and HAS3, using the UDP‐GlcNAc and UDP‐GlcUA as precursors. Generally, high‐molecular weight HA (HMW‐HA) is predominant in normal tissues. During tumor initiation and progression, HMW‐HA is rapidly degraded by hyaluronidases (HYAL) into low‐molecular weight HA (LMW‐HA) and small fragments of different lengths, which may possess pro‐inflammatory and pro‐angiogenic functions, promoting tumor growth, metastasis, and therapy resistance. This figure was created with BioRender.com.

Hyaluronic acid is typically produced by tumor and stromal cells, and its molecular weight determines whether it plays pro‐tumorigenic or antitumorigenic roles. High‐molecular weight HA (HMW‐HA) is predominant in homeostatic tissues. It protects cells from mechanical damage and has anti‐inflammatory and antiproliferative properties, thus hampering any pro‐tumorigenic events [11]. During tumor initiation and progression, HA is rapidly degraded by hyaluronidases into small fragments of different lengths, which possess pro‐inflammatory and pro‐angiogenic functions promoting tumor growth, metastasis, and therapy resistance [12] (Fig. 1).

In this review, we will provide a comprehensive overview of the current knowledge on the role of HA in cancer chemotherapy and its underlying molecular mechanisms. We will also examine the status and future prospects of HA‐based cancer therapy, highlighting the opportunities and challenges in this field.

## 2 Biology of HA

### 2.1 Basic information about HA

Hyaluronic acid is a glycosaminoglycan composed of repeating disaccharide units of d‐glucuronic acid (GlcUA) and *N*‐acetyl‐d‐glucosamine (GlcNAc) linked by glycosidic bonds. According to the length of disaccharide units, HA molecular weight ranges from 10^3^ to 10^7^ Da with a variable length of 2–25 μm. The purification of HA was first reported in 1934 when Karl Meyer and John Palmer isolated HA from the vitreous body of the bovine eye [13] while its structure was resolved later on in 1970. HA is present in many bacteria strains and is ubiquitous among all vertebrates, where it is particularly rich in embryonic tissue, for example, umbilical cord, and in adult soft connective tissues such as synovial fluid and vitreous body.

A prominent physico‐chemical property of HA is its capacity to absorb water up to 1000 times its molecular weight. Therefore, HA acts as a biological lubricant of the joints, keeping moisture retention and reducing friction [14], and hydrates the ECM, reducing tissue compression. HA can be produced with a variable molecular weight and different tissues and fluids might contain HA with different size.

Generally, HMW‐HA (1000–6000 kDa) is mainly synthesized under normal conditions and healthy tissues such as the epidermis contain high amount of HMW‐HA [15]. Low‐molecular weight HA (LMW‐HA, ≤ 250 kDa) are physiologically present at low level in fluids such as milk, blood, saliva [16, 17]. The ratio of these different HA species does not stay invariant and HA polymers with different lengths inside the HA mixture are dynamically regulated in response to physiological and pathological conditions. For instance, during cancer progression or during wound healing, HMW‐HA is progressively degraded by hyaluronidase enzymes (HAases, see Section 2.3) into LMW‐HA, in order to sustain the inflammatory phase [18, 19]. The dynamic regulation of HA metabolism suggests that distinct species of HA could modulate distinct biological outcomes. Indeed, the cellular response activated by HA species largely depends on their specific size and the cell types the HA fragments interact with. Generally, HMW‐HA is involved in maintaining tissue integrity and has antiproliferative, antiangiogenic, and immunosuppressive properties [12, 20, 21, 22, 23, 24, 25] (Fig. 1). However, HMW‐HA can exert also a pro‐proliferative function in vascular muscle cells and fibroblasts [26, 27]. LMW‐HA can also modulate proliferation and inflammation depending on cell type and the specific molecular size of the LMW‐HA species. For instance, LMW‐HA with a 35‐kDa weight induces breast cancer cell migration and invasion while a 117‐kDa HA fragment shows the opposite effects [28]. Regarding the inflammation response, LMW‐HA is widely recognized to be an important mediator of the inflammatory response [24]. However, several studies also suggest that small HA molecules can attenuate the inflammatory process and induce pro‐resolving responses [29]. Therefore, the precise biological outcome exerted by HA species is quite complex and can be cell‐type dependent.

In the next paragraphs, we will describe how the synergistic activity of biosynthetic and degradation processes together with the activity of specific HA receptors modulate HA continent and its biological outcomes.

### 2.2 HA regulation

In mammals, HA is synthesized by one of three distinct transmembrane hyaluronan synthases (HAS): HAS1, HAS2, and HAS3. These enzymes utilize UDP‐GlcNAc and UDP‐GlcUA as precursors to catalyze the synthesis of HA polymer chain, which is then transferred into the extracellular space through the pores in HAS structures along with its elongation [30]. Some studies have also shown that HA might be also secreted by the multidrug transfer protein in vertebrate cells [31, 32]. Although the three HAS enzymes have a similar structure and share 50–70% of similarity in their amino acid sequence, they possess specific enzymatic properties and are differently expressed during morphogenesis and pathological conditions.

HAS1 exhibits a slower rate for HA synthesis and produces the smallest polymer (0.12 × 10^6^ Da). Conversely, HAS2 produces the largest HA polymers (over 3.9 × 10^6^ Da) and accounts for the majority of cellular HA production. Accordingly, *Has2* ^−/−^ mice have severe cardiac and vascular abnormalities and die during midgestation. Conversely, *Has1* ^−/−^ and *Has3* ^−/−^ animals are viable and fertile, indicating that HAS2 is the only HAS enzymes required during embryogenesis [33]. HAS3 is the most active enzyme and produces intermediate‐length HA (0.12–1 × 10^6^ Da) [23].

HAS enzymes are differentially expressed in human tissues. HAS1 and HAS2 are highly expressed in adipose tissue, whereas HAS3 expression level is relatively abundant in the esophagus [34]. In mouse embryo, HAS2 is most abundant in mesenchymal tissues and heart while HAS3 staining is relatively low in mouse embryonic tissues [35]. However, it is worth noting that the lack of reliable antibodies against HAS proteins together with their low expression levels in many cell types may limit our knowledge on HAS's tissue distribution.

In several cancers, HAS expression correlates with cancer grade and can predict patient survival. In breast cancer cells, HAS1‐3 levels in stromal and malignant cells are related to tumor aggressiveness and poor patient outcome [36]. Similarly, in colon and ovarian cancers, increased HAS1 mRNA expression and protein correlates with poor survival of patients [37].

HAS expression is regulated both at transcriptional and post‐transcriptional levels and distinct growth factors and cytokines as well as glycolytic metabolites modulate HAS expression and activity in response to microenvironment changes (Fig. 1). At transcriptional level, various transcription factors interact with the promoter regions of HAS genes to influence their transcription and subsequent expression. HAS2 promoter region is characterized by binding sites for several transcription factors such as CREB1, retinoic acid receptor (RAR), SP1, STAT1, YY1, and FOXOs [38, 39]. Similarly, HAS1 expression is regulated by the transcription factors Smad3 and SP3 [40], while HAS3 is influenced by factors such as CEBP, SP1, and NFκB [41]. In head and neck squamous cell carcinoma (HNSCC), the transcription factor ΔNp63, a member of the p53 family that is widely expressed and deregulated in cancer [42, 43, 44, 45, 46, 47, 48, 49], binds to a specific p63‐binding site (p63 BS) in the promoter region of the HAS3 gene. This binding event enhances the expression of HAS3, thereby modulating the amount of HA synthetized by HNSCC cells [50, 51].

Post‐translational modifications include O‐GlcNAcylation of HAS2 and HAS3 and protein ubiquitylation of HAS2, which modulates enzymatic activity and protein stability, respectively [52, 53, 54]. The availability of HA precursors (UDP‐GlcUA and UDP‐GlcNAc) controls the levels of HA synthesis [55]. In addition, UTP, ATP, and their degradation products released from stressed cells can cause a transient upregulation of HAS expression [56, 57, 58]. Conversely, during energy restriction HAS2 is inactivated by a mechanism involving AMPK [59]. Different growth factors and cytokines, such as keratinocyte growth factor, epidermal growth factor, transforming growth factor‐β, interleukin‐1β or interferon‐c, and hormones (e.g., prostaglandins, corticosteroids, and retinoids) present in TME influence HAS expression [38].

### 2.3 HA catabolism

Under normal conditions, the synthesis and degradation of HA are tightly regulated. Pathological conditions, such as cancer and inflammation, promote extensive HA remodeling, mainly by inducing endogenous HMW‐HA degradation by hyaluronidases, resulting in increased levels of LMW‐HA species.

There are seven different hyaluronidases (HAases) in humans: HYAL1, HYAL2, HYAL3, HYAL4, PH‐20, Hyalp‐1, and HYBID [60, 61]. Among these HAases, the most studied are HYAL1, HYAL2, and PH‐20. HYAL1 and HYAL2 are widely expressed in most tissues. HYAL2 is a glycosyl phosphatidyl‐inositol (GPI)‐anchored cell surface enzyme able to bind to and internalize HA into vesicles, where it catalyzes the digestion of HA into 20 kDa fragments. The 20 kDa HA fragments are subsequently internalized into the lysosome where HYAL1 further processes them into oligosaccharides, which eventually can be further degraded into monosaccharides by exoenzymes [62]. PH‐20, the most active mammalian hyaluronidase, is necessary for fertilization of the oocyte by sperm and possesses signaling properties [63].

HA fragments of different sizes may also be generated by reactive oxygen species (ROS) or reactive nitrogen species (RNS), mainly during inflammatory processes and tissue repair [64]. ROS‐generated HA species are molecularly distinct from those raising from the activity of HAases since they are generated randomly with a polydisperse size. ROS‐mediated degradation of HA is mainly involved in the wound healing process as it enhances the inflammatory response which in turn stimulates HA synthesis and dermal fibroblasts proliferation with the subsequent formation of a new ECM [65, 66].

HAase expression is generally elevated in a variety of carcinomas and is considered a tumor biomarker. However, in some carcinomas, HAase expression is inversely correlated with tumor grade [67]. For instance, higher HYAL2 expression is associated with poor prognosis of triple‐negative breast cancer patients and silencing of HYAL2 expression reduces tumorigenicity in a tumor xenograft model [68]. In terms of tumor treatment strategies, since HA accumulation is often observed in the tumor stroma where it contributes to cancer progression, PEGylated human recombinant PH20 hyaluronidase (PEGPH20) has been developed as an anticancer therapy and is currently in clinical trials [69]. We will describe this HA‐based approach in Section 4.

### 2.4 HA receptors

HA receptors are ubiquitously expressed in a variety of different cell types, such as endothelial cells, epithelial cells, and immune cells. The main HA receptors include the cluster of differentiation 44 (CD44), the receptor for hyaluronan‐mediated motility (RHAMM), the HA receptor for endocytosis (HARE), the lymphatic vessel endothelial hyaluronan receptor 1 (LYVE‐1), toll‐like receptor 2 and 4 (TLR‐2 and TLR‐4) and layilin. Here, we briefly describe CD44 and RHAMM since evidence have demonstrated their crucial involvement in the tumor pathogenesis (for an extensive review on HA receptors, please see [70]).

CD44, a nonkinase transmembrane glycoprotein, acts as an important signaling hub that initiates diverse intracellular signaling cascades affecting multiple processes (e.g., survival, inflammation, cytoskeleton remodeling and motility, and epithelial to mesenchymal transition). CD44 gene undergoes complex alternative splicing events which generate multiple variants such as CD44v3 and CD44v6 isoforms, which are critically involved in regulating cancer stem cell homeostasis contributing thus to tumor chemioresistance [71, 72]. CD44 proteins have distinct structural domains that include an N‐terminal HA‐binding link‐homology module, which is responsible for HA interaction, a stem region, a transmembrane domain, and a short C‐terminal cytoplasmic domain, which is responsible for the activation of intracellular signaling pathways upon HA‐CD44 interaction [73]. The downstream signaling routes activated by HA‐CD44 association are mediated by the activation of diverse cytoskeletal proteins and intracellular signaling components, such as the Rho‐family GTPases, Src family kinases, and associated molecules, which play a role in regulating cell adhesion and migration [74].

In addition to initiate intracellular signaling, CD44‐HA interaction might also modulate the activity of growth factor receptors. At functional level, HA‐CD44 interaction mediates cell proliferation and survival of different cancer cells, mainly through the activation of the EGFR/AKT/ERK signaling pathway [75]. In breast cancer [76] and HNSCC cells [77, 78], HA‐CD44 interaction promotes chemoresistance by enhancing c‐Jun signaling pathways and microRNA‐21 expression. Furthermore, HA‐CD44 interaction has been implicated in mediating epithelial–mesenchymal transition in many cancer cells [79, 80].

Similar to CD44, RHAMM, also termed CD168, encodes alternative splicing isoforms (e.g., RHAMM^A^ and RHAMM^B^). RHAMM is expressed at low levels in normal tissues and its expression is transiently induced upon specific signals or during several biological processes, such as wound repair [81]. Interestingly, recent evidence demonstrated that RHAMM is more sensitive to the density of HA than CD44 in breast cancer cell lines, and its surface expression can be enhanced to compensate the pro‐tumorigenic signaling mediated by CD44 when CD44 is blocked [82]. In various carcinomas (e.g., colorectal cancer [83], pancreatic ductal adenocarcinoma (PDA) [84], breast cancer [85], and multiple myeloma [86]), RHAMM is overexpressed and commonly associated with poor prognosis [87, 88]. At the functional level, in tumor cells RHAMM‐HA interaction elicits multiple signaling cascades that regulate the following tumor‐related features: (a) it enhances cell motility and invasion, mainly by activating protein kinase C (PKC) [89, 90, 91]; (b) it boosts cell proliferation via regulating the expression of mitogen‐activated protein kinase (MAPK) [85], and (c) it induces epithelial‐to‐mesenchymal transition and multidrug resistance by increasing TGFβ/smad2 expression [92].

## 3 HA‐mediated chemoresistance in tumors

**Fig. 2:**
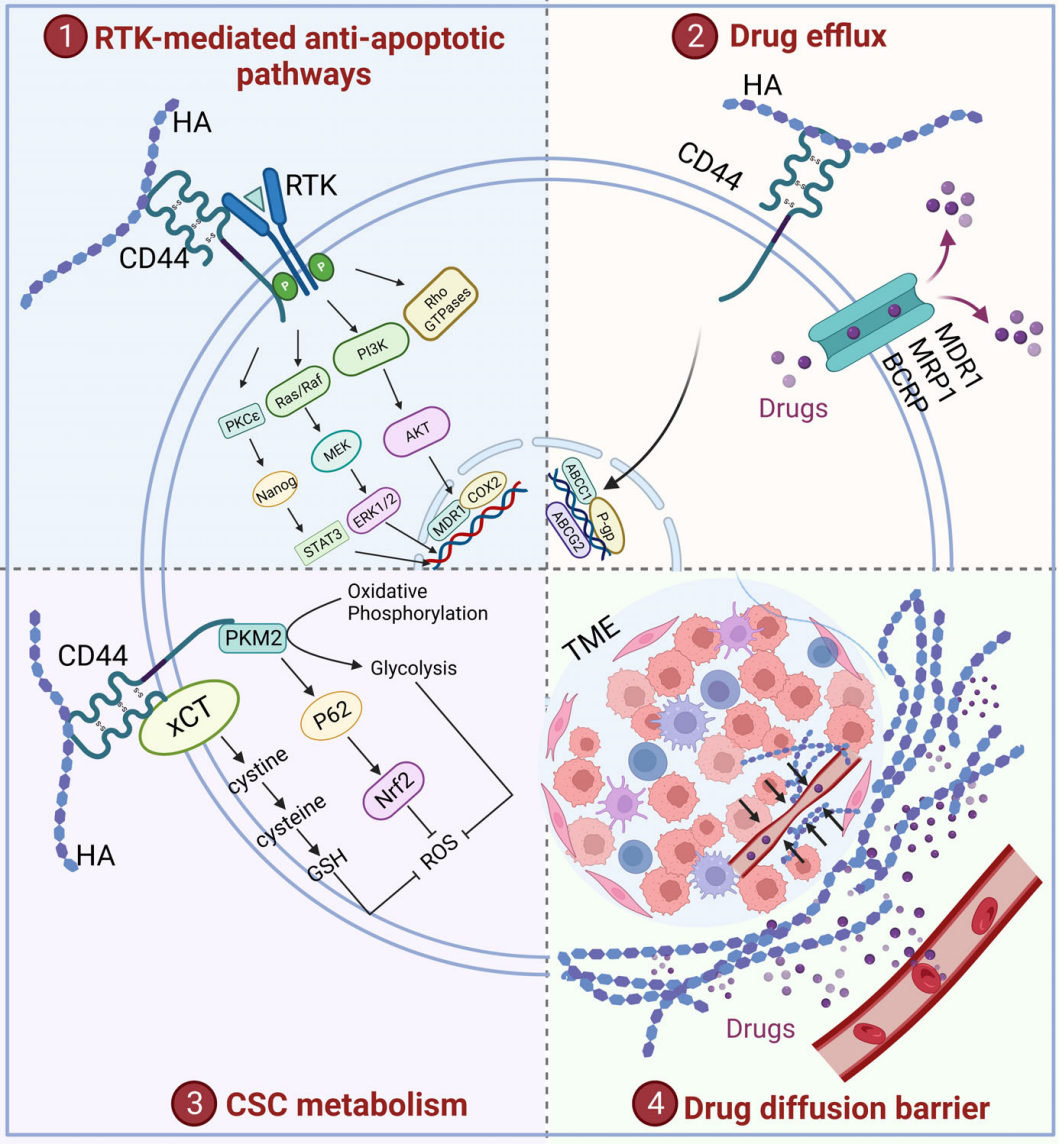
Mechanisms of hyaluronic acid (HA)‐mediated chemoresistance in tumors. (1) CD44 regulates receptor tyrosine kinase (RTK) activation and modulates different antiapoptotic signaling pathways, which promote chemoresistance. (2) HA‐CD44 interaction regulates multidrug resistance in cancer cells. (3) HA‐CD44 axis regulates cancer stem cell (CSC) properties through induction of oxidative stress resistance. (4) HA acts as a biophysical barrier impairing vascular function and drug delivery in tumor cells. This figure was created with BioRender.com.

HA is a significant component of TME and influences tumor development and prognosis [93]. The involvement of HA in tumor chemoresistance is mainly mediated by the CD44 receptor. The interaction between HA and CD44 promotes antiapoptotic pathways induced by receptor tyrosine kinase (RTK) activation, enhances transporter activities, and confers cancer stem cells (CSCs) with resistance to oxidative stress. Additionally, HA, as biophysical barrier, impairs vascular function and drug delivery, further contributing to chemotherapy resistance (Fig. 2). Understanding the molecular mechanisms by which HA mediates chemotherapy resistance can provide insights into novel therapeutic strategies to overcome resistance and improve drug sensitivity in chemotherapy‐tolerant patients.

### 3.1 HA regulates receptor tyrosine kinases activation and antiapoptotic signaling pathways

Enhanced activation of cell survival/antiapoptotic pathways is a common phenomenon in tumors and significantly contributes to drug resistance [94]. By acting as plasma membrane receptors of growth factors, RTKs activate antiapoptotic signal transduction pathways [95]. Many studies have demonstrated that HA is able to modulate RTK activation, thus eliciting prosurvival and antiapoptotic signaling. In detail, the following intracellular cascades are influenced by HA‐mediated signaling:

#### 3.1.1 Phosphoinositide 3‐Kinase (PI3K) pathway

In breast cancer cells, a positive feedback loop coupling CD44 splice isoform and HAS2 results in increased PI3K/Akt activation which ultimately leads to cell death resistance [96]. In colon and breast carcinoma cells, elevated levels of HA result in the formation of a signaling complex involving CD44 and ErbB2, along with PI3K, ezrin, HSP90, and CDC37. This complex leads to the activation of the AKT pathway and stimulates the expression of the multidrug resistance gene MDR1 [97, 98]. In HA overexpressing intestinal epithelial and colon carcinoma cells, HA‐CD44 interaction activates ErbB2‐PI3K/AKT‐β‐catenin signaling and enhances tumor cell growth and survival by inducing COX‐2 expression [99]. In HNSCC, HA‐CD44 promotes Rho kinase‐ and PI3K‐mediated oncogenic signaling and cisplatin resistance [100].

#### 3.1.2 MAPK pathway

The MAPK pathway is a crucial signal transduction pathway that involves the activation of RAS GTPase. This activation leads to the activation of downstream kinases, including RAF, MEK, and extracellular response kinase 1 and 2 (ERK1 and ERK2), which regulate cell survival, proliferation, differentiation, and apoptosis [101]. In HNSCC, HA‐CD44 promotes EGF‐R activation, which in turn activates ERK1 and 2 to promote tumor cell growth, migration, and chemoresistance [102]. Another study demonstrated that in breast cancer cell lines, HA/CD44/RHAMM forms a signaling complex with ERK‐1,2 to sustain tumor invasiveness [103].

#### 3.1.3 Rho GTPase signaling

Rho GTPases are a family of small GTP‐binding proteins that act as molecular switches inside cells, regulating various cellular processes such as cytoskeletal organization, cell migration, proliferation, and gene expression. The Rho GTPases family includes several members, including Rho, Rac, and Cdc42, which are the most extensively studied [104]. In cancer cells, Rho GTPase signaling promotes cell motility via actin polymerization, which can affect cell stiffness and mediate chemoresistance [105]. Several lines of evidence suggest that HA‐CD44 association might mediate the activation of Rho GTPase signaling and cytoskeleton rearrangement, which in turn can promote tumor progression and chemoresistance [106]. For instance, in HNSCC cells, HA‐CD44 mediates Rho GTPase activation by a DOT1L/H3K79‐dependent mechanism and confers cisplatin resistance [100, 107].

#### 3.1.4 Other HA‐mediated antiapoptotic signaling pathways

In addition to regulating RTK signaling, HA activates other anti‐apoptotic signaling pathways. In breast tumor cells, HA‐CD44 promotes PKCepsilon activation, which induces Nanog nuclear translocation. Nuclear Nanog associates with RNase III DROSHA and the RNA helicase p68, leading to induction of microRNA‐21 expression and repression of the pro‐apoptotic protein PDCD4. These events ultimately promote tumor cell survival and chemotherapy resistance [108]. HA‐CD44‐mediated Nanog‐Stat‐3 signaling pathways have a similar effect in ovarian tumor and breast tumor cells [109]. The HA receptor‐ RHAMM confers resistance to 5‐FU via TGFβ/Smad2‐induced EMT in gastric cancer [92]. HA‐CD44 also regulates the Hippo signaling pathway, which modulates tumor cell death and proliferation [68, 110].

Therefore, targeting HA‐CD44‐mediated signaling by disruption of the HA‐CD44 complex (see Section 4) might be an effective strategy to dampen tumor chemoresistance.

### 3.2 HA‐mediated multidrug resistance

Tumor chemoresistance is the result of diverse molecular pathways. One of the most important mechanisms underlying tumor drug resistance is related to the increased efflux rate of antineoplastic drugs by members of the ATP‐binding cassette (ABC) transporters family. There are 48 members of this protein family, of which the most extensively studied are MDR1 (also known as P‐glycoprotein or ABCB1), MRP1 (also known as ABCC1), and BCRP (also known as ABCG2) [111].

MDR1 (P‐gp, ABCB1) was the first ABC transporter to be identified. It is overexpressed in many solid tumors and its expression can be induced by chemotherapy, thus causing intrinsic and acquired chemoresistance [112]. In breast cancer cells, a positive feedback loop involving HA, PI3K, and ErbB2 strongly amplifies the expression of MDR1 and increases resistance to doxorubicin. This HA‐mediated effect has also been reported in another study showing that HA‐CD44 stimulates MDR1 expression and induces resistance to doxorubicin, paclitaxel, and etoposide [108, 109, 113].

Similar to MDR1, MRP1 (ABCC1), the second member of the ABC transporter family to be identified [114], has been correlated with chemoresistance in many types of solid cancers. As mentioned before, in HNSCC cells the oncogenic activity of transcription factor ΔNp63 sustains the HA levels and signaling by transcriptionally regulating the expression of HAS3, HYAL‐1, HYAL‐3, and CD44. This ΔNp63‐driven pathway leads to the HA‐dependent activation of EGF‐R and the induction of ABCC1 expression. Notably, in HNSCC tumors p63 expression is positively correlated with the expression of CD44, HAS3, and ABCC1 while the p63‐HA pathway acts as a negative prognostic factor for the survival of HNSCC patients [50]. Similar to ABCC1, ABCC2 expression is also modulated by HA. In ovarian cancer, carboplatin significantly increases the expression of HAS2, HAS3, and ABCC2. Along the same line, in nonsmall cell lung cancer cells HA promotes the expression of ABCC2 and induces resistance to cisplatin [115]. Furthermore, HA treatment markedly induces the expression of ABCB3, ABCC1, ABCC2, and ABCC3 [116].

BCRP (ABCG2) was the third MDR drug efflux pump to be identified. In malignant gliomas, HA oligomers inhibit activation of EGFR and AKT, decrease BCRP expression, and increase methotrexate cytotoxicity [117, 118].

In addition to regulating the expression of ABC members at the transcriptional level, HA‐mediated signaling might also impact ABC transporter expression by stabilizing their levels in the plasma membrane, thereby enhancing their activity [119]. In malignant peripheral nerve sheath tumor cells, CD44 interacts with BCRP and P‐glycoprotein and HA oligomers treatment induces internalization of CD44, BCRP, and P‐glycoprotein, leading to decreased plasma membrane localization of drug transporters and enhanced sensitivity to doxorubicin [118]. Another study also confirmed the physical and genetic interaction between CD44s and P‐glycoprotein, which results in invasion and multidrug resistance in cancer cells [119].

Another interesting point about HA and ABC transporters family is that HA might be secreted through multidrug transporters in vertebrate cells [31]. Thus, drugs that inhibit multidrug transporters may also inhibit HA secretion and could represent an alternative strategy to dampen HA‐mediated signaling.

### 3.3 Regulation of cancer stem cell properties by HA‐CD44 signaling

CSCs are a subpopulation of cells that possess stem cell‐like characteristics, such as self‐renewal and differentiation potential, and are thought to be responsible for tumor initiation, maintenance, progression, and therapeutic resistance [120, 121]. In human cancers, CSCs have been identified through different biomarkers. CD44 is a marker of CSCs in almost all tumors and, as primary receptor of HA, has a significant impact on the characteristics of CSCs including resistance. For instance, in ovarian cancer and breast cancer cells, HA interacts with CD44 to promoting the formation of a complex between CD44, Nanog, and STAT‐3, which in turn induces the expression of SOX2, REX1, and MDR1, enhancing thus resistance to doxorubicin and paclitaxel [109]. In HNSCC, HA significantly promotes the upregulation of a subset of antiapoptotic proteins and, as a consequence, CSCs resistance to cisplatin [122]. Here, we mainly describe how the HA‐CD44 axis affects CSC therapeutic resistance through oxidative stress resistance.

The buildup of ROS triggers apoptosis in both normal and cancer cells. CSCs are characterized by upregulation of several antioxidant pathways able to maintain low ROS intracellular levels [123, 124, 125]. Therefore, CSCs are relatively more resistant to radiation‐ or chemotherapy‐induced cell death compared with nontumor cells [126]. One of the main antioxidant pathways upregulated in CSCs is mediated by the nuclear factor erythroid 2‐like 2 (NFE2L2; NRF2), a transcription factor able to induce the expression of key antioxidant genes [127, 128, 129]. NRF2 is highly expressed in CD44^high^CD24^low^ CSC subpopulation isolated from doxorubicin‐resistance breast cancer cells. CD44 overexpression induces the deregulation of hypoxia, upregulation NRF2, and HA itself can enhance NRF2 activation. Functionally, the molecular mechanism of CD44‐mediated NRF2 activation involves high expression of p62 [130]. Once activated, NRF2 induces the expression of many genes encoding multiple antioxidant proteins, for example, glutathione (GSH)‐generating enzymes. In turn, CD44 interacts with xCT, a subunit of a glutamate‐cystine transporter, thereby promoting the uptake of cystine for GSH synthesis. In human gastrointestinal cancer cells, high level of CD44 expression is associated with an enhanced capacity of GSH synthesis and defense against ROS. These findings suggest that CD44‐targeted therapy may impair the ROS defense ability of CSCs thereby sensitizing them to cancer therapy [131]. Similar results were also demonstrated in urothelial [132] and triple‐negative breast cancer cells [133]. Another mechanism whereby CD44 enhances cellular defense against ROS‐mediated cellular damage in CSCs is the regulation of glucose metabolism. By performing a proteomic screen of the binding proteins of CD44, it was discovered that pyruvate kinase M2 (PKM2) binds to the C‐terminal tail of CD44 [134]. The interaction between CD44 and PKM2 enhances the glycolytic phenotype of cancer cells. CD44 ablation shifts tumor cell metabolism toward mitochondrial oxidative phosphorylation, resulting in increased ROS production, decreased level of the intracellular GSH, and higher sensitivity to ROS‐mediated antineoplastic drugs [135].

### 3.4 HA as biophysical barrier impairs vascular function and drug delivery

Pancreatic ductal adenocarcinoma is one of the human malignancies whose TME is characterized by vascular dysfunction and extensive ECM deposition. HA is highly abundant in the ECM of PDA, and mediates PDA chemoresistance mainly by physical mechanisms [136]. Provenzano et al. [136] showed that HA significantly increases the interstitial fluid pressure (IFP) in PDA and induces vascular collapse, creating substantial barriers to perfusion, diffusion, and convection of small molecule drugs. By using a clinically formulated PEGylated human recombinant PH20 hyaluronidase (PEGPH20) to deplete HA, the authors showed that HA degradation significantly impacts IFP leading to a dramatic increase in vessel diameter, which in turn allows high concentrations of chemotherapy to reach the tumor, thereby improving the cytotoxic effects of the antineoplastic drugs. Similar evidence has also been provided by Jacobetz et al. [137]. The authors found that depletion of HA by PEGPH20 induces the re‐expansion of PDA blood vessels and the increase in the delivery of two chemotherapeutic agents, doxorubicin, and gemcitabine. In addition, PEGPH20 induces ultrastructural changes in tumor endothelial cells, leading to a significant increase in the fenestration of the endothelial barrier, which is a phenotype associated with high permeability. Furthermore, the combination therapy of PEGPH20 and gemcitabine inhibits the growth of PDA tumors and prolongs survival compared with gemcitabine monotherapy. Similar results have also been obtained in prostate cancer. In high‐HA prostate PC3 tumors, administration of PEGPH20 via intravenous injection results in depletion of tumor HA, reduction in tumor stromal pressure and water content, decrease in the tumor vascular pressure, and more than a threefold increase in tumor vascular area. Moreover, PEGPH20 boosts the efficacy of docetaxel and liposomal doxorubicin in PC3‐derived tumors [138]. Based on these preclinical observations, PEGPH20 efficacy and toxicity have been evaluated in clinical trials. We will provide a detailed overview of these findings in the following sections.

In addition to affect drug delivery and chemotherapy efficacy, HA can directly bind to doxorubicin and form strong interactive forces, which hinders its cellular entry in the tumor tissue [139].

## 4 Potential applications of targeting HA in cancer therapy

**Fig. 3:**
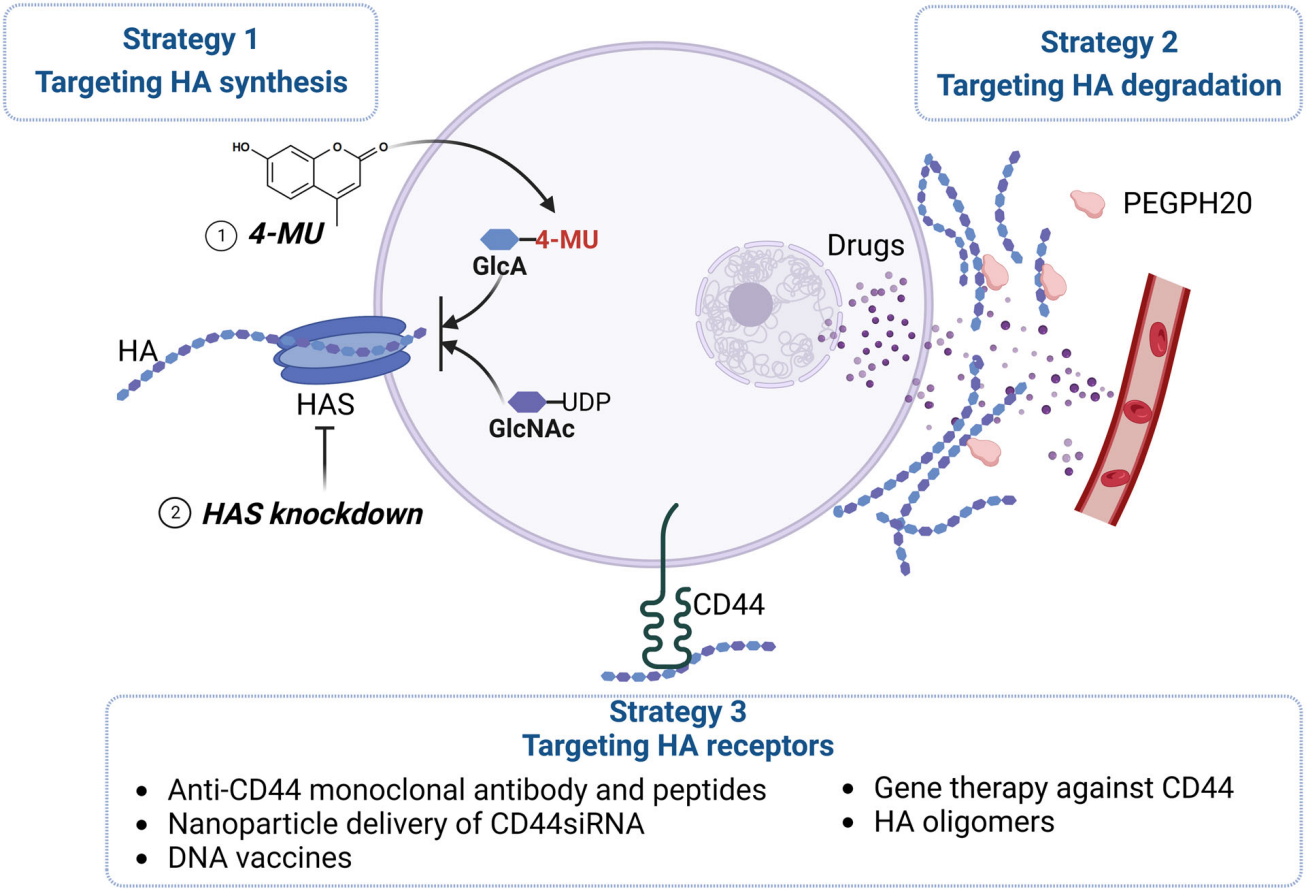
Potential applications of targeting hyaluronic acid (HA) in cancer therapy. Three strategies have been employed to target HA in cancer: targeting HA synthesis (1), targeting HA degradation (2), and targeting HA receptors (3). This figure was created with BioRender.com.

The role of HA in promoting tumor chemoresistance suggests that HA is a valuable therapeutic target for treating cancer and enhancing the efficacy of chemotherapy. Several strategies have been employed to target HA in cancer, including targeting HA synthesis, degradation, and its interaction with the receptors (Fig. 3).

### 4.1 Targeting HA synthesis

Given the contributions of HA and its synthesis to tumor progression and chemotherapy tolerance, there is significant interest in finding pharmacological methods to impede HA synthesis. So far, the only available inhibitor of HA synthesis is 4‐methylumbelliferone (4‐MU). 4‐MU, a coumarin derivative, is present in various Chinese herbal medicines. 4‐MU is also called ‘hymecromone’, and it is clinically utilized to treat biliary spasm in Europe and Asia [140].

4‐MU inhibits HA synthesis by two different mechanisms. Firstly, 4‐MU is a competitive substrate for UDP‐glucuronosyltransferase (UGT), an enzyme involved in HA synthesis [141]. Secondly, 4‐MU reduces the expression of HAS mRNA expression [142]. In agreement with the critical role of HA metabolism in controlling tumor behavior, 4‐MU treatment exerts antitumorigenic effects in multiple cancer cell types mainly by an HA‐dependent mechanism [143, 144, 145, 146, 147, 148, 149]. For example, in glioblastoma cells, the ability of 4‐MU to decrease HA synthesis, proliferation, cell migration, and induce apoptosis is dependent on HA [146].

Treatment with 4‐MU can also enhance the efficacy of chemotherapy and immunotherapy. In ovarian cancer, 4‐MU inhibits HA production and tumor cell survival in both chemo‐resistant and chemo‐sensitive cells. In chemotherapy‐resistant tumor cells, the combination of 4‐MU and carboplatin inhibits cell survival more effectively than carboplatin alone. This result has also been validated by using an *ex vitro* explant assay demonstrating that the combined treatment of 4‐MU with carboplatin significantly increases apoptosis and reduces proliferation in tissues from chemo‐resistant patients [150]. In malignant pleural mesothelioma, the combination of trametinib and 4‐MU treatment has a greater inhibitory effect in comparison to trametinib monotherapy [151]. 4‐MU also boosts the antitumor effect of immunotherapy. In murine colorectal carcinoma, 4‐MU significantly reduces the level of tumoral HA, decreases the tumor interstitial pressure, and also increases the number of cytotoxic T lymphocytes reaching the tumor [152]. Furthermore, antitumoral efficacy of cyclophosphamide + interleukin‐12 is improved by 4‐MU [152]. Several clinical trials have also demonstrated the excellent safety profile of 4‐MU during short‐term administration [153, 154]. Thus, 4‐MU has the potential to be an effective agent for cancer inhibition by enhancing the efficacy of chemotherapy and immunotherapy.

It is worth noting that other evidence indicates that 4‐MU might exert its antitumor effect independently of HA [155, 156]. For instance, 4‐MU inhibits the growth of ovarian cancer cells by suppressing thymidine phosphorylase expression [156]. Furthermore, 4‐MU may impact the synthesis of additional ECM components, such as versican and fibronectin, and other glycosaminoglycans, such as chondroitin and heparin sulfates [157, 158]. During the ECM remodeling induced by liver fibrosis, the synthesis of collagen 1A is also affected by 4‐MU treatment [159]. In addition to ECM components, 4‐MU can regulate the activity of the matrix metalloproteinases (MMPs), a family of enzymes capable to degrade many ECM components leading to tissue degradation and remodeling in an HA‐independent manner [160, 161]. Notably, the action of 4‐MU on MMPs is different when comparing normal vs tumor tissue. More specifically, in human skin fibroblasts, 4‐MU induces MMP2 activation [162], while in human carcinoma cells, 4‐MU inhibits MMP9 [163]. Altogether, these studies suggest that although the main action exerted by 4‐MU is HA synthesis inhibition, other HA‐independent effects of 4‐MU need to be considered.

In addition to dampen tumor progression, 4‐MU has been recently utilized as a potential drug for COVID‐19 therapy. Lungs of critically ill patients with COVID‐19 infection are characterized by the presence of clear liquid jelly mainly consisting of HA [164]. By reducing the accumulation of HA and favoring the clearance of the jelly, 4‐MU might restore proper alveoli function, reducing the need of ventilators for patient care [165]. This hypothesis has also been validated in clinical trials [166].

Another approach to dampen HA synthesis is by inhibiting HAS expression. There are many studies that have shown that HAS knockdown can induce apoptosis, inhibiting both tumor growth and angiogenesis in multiple cancer types [153].

### 4.2 Targeting HA degradation

Targeting the HA catabolism process has also been proposed to prevent HA accumulation in tumor cells and improve the efficacy of chemotherapeutic agents. HYAL (mainly PEGPH20) has been evaluated in several clinical trials in combination with other therapies (such as chemotherapy, immunotherapy, or radiotherapy) in different types of cancer [167, 168, 169, 170, 171, 172, 173, 174] (Table 1). In an early study of 40 pediatric brain cancer patients, the combination of HYAL with standard chemotherapeutics (carboplatin and etoposide) significantly improved event‐free survival and overall survival [172]. This reduced recurrence was also seen in a study of HNSCC [170]. In 2013, the safety, pharmacokinetic, and pharmacodynamic profile of PEGPH20 in patients with a variety of solid tumors was demonstrated in a phase I clinical study. The results demonstrate a reduction in HA levels within the tumor, improved tumor perfusion, and decreased tumor metabolic activity. These findings provide support for further evaluation of the combination of PEGPH20 with anticancer therapies in patients with advanced solid tumors [69]. In 2016, Hingorani et al. [168] reported the results of a phase 1b study of PEGPH20 in combination with gemcitabine to treat stage IV metastatic PDA. According to clinical trial, the combination of PEGPH20 and gemcitabine is well tolerated and could be an effective treatment option for patients with advanced PDA, particularly those with high levels of HA in tumors. In 2018, they reported the results of a phase II study of PEGPH20 in combination with nab‐paclitaxel/gemcitabine (PAG) in 279 patients with metastatic PDA. PEGPH20 combination therapy consistently improved the progression‐free survival (PFS; overall) [169]. Although these phase I/II results were encouraging, the subsequent HALO‐301 phase III trial did not meet its primary endpoint of OS [175, 176]. In detail, this study, which enrolled patients with stage IV PDA expressing high amount of HA, compared PEGPH20 in combination with PAG to PAG alone. No improvement in duration of response, PFS, or OS was seen in PEGPH20‐treated patients. The failure of this phase III study together with the high toxicity observed in the PEGPH20‐FOLFIRINOX (folinic acid fluorouracil irinotecan oxaliplatin) combination [177] suggest that targeting HA might not be sufficient for eradicating tumor cells. Other intrinsic factors such the high complexity of TME and the low tumor mutational burden may considerably concur to the elevated chemoresistance of PDA. Furthermore, it is worth noting that disruption of the tumor stroma might also facilitate the metastatic capacity of tumor cells, as described for PDA deficient in Sonic Hedgehog signaling [178]. Therefore, further research and evaluation need to be directed to understand how the complex interplay between HA dysregulation, TME complexity, and metastatic potential in order to increase the efficacy and safety of HA‐based drugs.

**Table 1:** Clinical trials of hyaluronidase‐targeted therapies.

Study	Trial type	Tumor type	Chemotherapy	No. of patients	Results
Smith KJ et al., 1997, (124)	Phase I	Kaposi's sarcoma	Vinblastine	6	Enhances drug efficacy and reduces recurrence
Pillwein K et al., 1998 (123)	Phase II	Malignant brain	Carboplatin/etoposide	40	Event‐free survival and overall survival at 36 months were significantly improved
Klocker J et al., 1998, (125)	Phase II	Advanced squamous cell carcinoma of the head and neck	Irradiation + cisplatin/vindesine	48	The disease‐free survival rate at 5 years was 47%
Baumgartner G et al., 1998, (126)	Phase III	Bladder cancer	Mitomycin C	56	Reduced recurrence
Hingorani SR et al., 2016, (102)	Phase Ib	Pancreatic ductal adenocarcinoma	Gemcitabine	28	PEGPH20 in combination with Gem was well tolerated and has therapeutic benefit
Hingorani SR et al., 2018, (103)	Phase II	Pancreatic ductal adenocarcinoma	Nab‐paclitaxel/gemcitabine	279	Progression‐free survival was significantly improved
Van Cutsem E et al., 2020, (127)	Phase III	Metastatic pancreatic adenocarcinoma	Nab‐paclitaxel/gemcitabine	494	Increased the ORR but did not improve OS or PFS
Ko AH et al., 2023, (128)	Phase Ib/II	Pancreatic ductal adenocarcinoma/gastric cancer	Atezolizumab	108	Atezolizumab plus PEGPH20 showed limited clinical activity in patients with PDAC and none in patients with GC

### 4.3 Targeting HA receptors

The interaction between HA and CD44, as we described above, modulates chemoresistance of tumors through multiple mechanisms. Therefore, CD44 is an attractive target to modulate HA‐dependent signaling. The therapeutic approaches aimed to interfere with CD44 signaling are mainly based on targeting either CD44‐HA interaction or CD44 itself through different strategies, such as antiCD44 monoclonal antibody [179], peptides [180], nanoparticle delivery of CD44 siRNA [181], DNA vaccines [182], or gene therapy against CD44 [183]. For example, monoclonal antibodies targeting CD44v6 have been demonstrated to be effective in hampering tumor growth in preclinical studies [180]. However, phase I clinical studies failed due to the severe skin reactions likely caused by the physiological expression of CD44v6 antigen in normal squamous cells [184]. Hence, the employment of the CD44 targeting approach in cancer therapy would require meticulous assessment before implementation [79, 184].

Alternative approaches aimed at blocking CD44‐mediated signaling include the usage of soluble CD44 or HA oligomers which can act as competitors of CD44 thus impeding HA‐CD44 interaction. In melanoma cells, soluble CD44 acts as a competitor of CD44 and blocks HA‐CD44 interaction, thereby inhibiting tumor growth and metastasis [185]. In the plasma membrane of malignant peripheral nerve sheath tumor cells, HA oligosaccharides disrupt the complex between CD44, BCRP (ABCG2), and P‐glycoprotein (ABCB1), thereby inhibiting drug transporter activity and increasing doxorubicin sensitivity [118]. HA oligomer treatment also significantly inhibits the growth of melanoma [186] and glioma tumor cells [117]. In HepG2iso cells and endothelial cells, HA oligomers inhibit cell motility and angiogenesis by interfering with HMW‐HA‐CD44 interaction [187]. It has also been reported that HA oligosaccharides impact on the anchorage‐independent tumor cell proliferation by suppressing the PI‐3K/Akt cell survival pathway [188]. Overall, these results demonstrate that HA oligomers might be potentially exploited as an approach to dampen HA‐mediated pro‐tumorigenic effects.

The interaction between HA and its receptor CD44, together with the excellent HA biocompatibility, biodegradability, and nonimmunogenicity, has also been exploited to increase the delivery of conventional antineoplastic drugs into tumor cells. HA can be either conjugated directly with antitumor drugs or used in several types of nanomaterials, such as micelles and hydrogels [189, 190].

A promising approach for treating colon cancer patients was based on the noncovalent conjugation of Irinotecan with a domain of HA. Although this approach successfully passed phase I and phase II trials [191, 192], multicenter phase III study failed to meet statistical significance between HA–Irinotecan‐based FOLFIRI (folinic acid, 5‐FU, and irinotecan) versus standard FOLFIRI in metastatic colon cancer patients [193]. The failure of this phase III study might be due to intrinsic factors such as the upregulation of drug efflux channels which might impact the intracellular concentration of irinotecan [194], or the decreases of CD44 expression which would render the tumor cells largely less responsive to CD44‐targeted treatment [195]. Alternatively, a more rigorous enrollment criterion based for instance on the analysis of CD44 expression levels could facilitate a better stratification of the patients and decrease patient heterogeneity.

## 5 Conclusion

Chemotherapy resistance represents a major cause of therapeutic failure and mortality in cancer patient. HA accumulation in the TME is able to promote either tumor growth, or recurrence and therapy resistance, according to distinct molecular circumstances. Mechanistically, HA acts as a pro‐chemo‐resistant factor by regulating multiple pathways, such as activation of RTKs and antiapoptotic signaling, induction of drug transporter expression, regulation of cancer stem cell properties and by acting as a barrier impairing vascular function and drug delivery. Hence, HA‐based strategies have been developed to enhance the effectiveness of chemotherapeutic agents and overcome chemoresistance in multiple types of cancers. Nevertheless, HA‐based cancer therapy faces challenges. HA synthesis and degradation is a complex circuit and the proportion of HA polymers with different lengths inside the HA mixture is dynamically regulated. Understanding the detailed proportion of HA polymers under certain circumstances is pivotal to predict the cellular response of HA dysregulation on a specific biological process. HA abundance and chain lengths dynamically remodel the mechanical and biological properties of the TME and affect the intracellular signaling cascades during tumor progression; it is important to understand HA homeostasis in a dynamic manner in order to better interpret HA‐mediated signaling. Furthermore, the detection of the serum concentration of specific HA fragments would provide a more meaningful prognosis predictor and diagnostic index in certain human diseases. In conclusion, future research effort should be focused on tackling these challenges to provide a better know‐how of the clinical application of HA‐based therapy.

## Conflict of interest

The authors declare no conflict of interest.

## Author contributions

ZL and AP wrote the manuscript. ZL prepared the figures. PH, JF, CS, and YS provide comments. GM revised the manuscript. All authors have approved this submitted version.

## References

[1] Fan T , Wan Y , Niu D , Wang B , Zhang B , Zhang Z , et al. Comprehensive analysis of pyroptosis regulation patterns and their influence on tumor immune microenvironment and patient prognosis in glioma. Discov Oncol. 2022;13:13. 35274175 10.1007/s12672-022-00474-5 PMC8913830

[2] Holohan C , Van Schaeybroeck S , Longley DB , Johnston PG . Cancer drug resistance: an evolving paradigm. Nat Rev Cancer. 2013;13:714–726. 24060863 10.1038/nrc3599

[3] Liu J , Cao X . Glucose metabolism of TAMs in tumor chemoresistance and metastasis. Trends Cell Biol. 2023;33:967–978. 37080816 10.1016/j.tcb.2023.03.008

[4] Mercanti L , Sindaco M , Mazzone M , Di Marcantonio MC , Piscione M , Muraro R , et al. PDAC, the influencer cancer: cross‐talk with tumor microenvironment and connected potential therapy strategies. Cancers (Basel). 2023;15:2923. 37296886 10.3390/cancers15112923 PMC10251917

[5] Ping S , Gong R , Lei K , Qing G , Zhang G , Chen J . Development and validation of a ferroptosis‐related lncRNAs signature to predict prognosis and microenvironment for melanoma. Discov Oncol. 2022;13:125. 36371574 10.1007/s12672-022-00581-3 PMC9653531

[6] Takasugi M , Yoshida Y , Ohtani N . Cellular senescence and the tumour microenvironment. Mol Oncol. 2022;16:3333–3351. 35674109 10.1002/1878-0261.13268 PMC9490140

[7] Ganini C , Amelio I , Bertolo R , Bove P , Buonomo OC , Candi E , et al. Global mapping of cancers: the cancer genome atlas and beyond. Mol Oncol. 2021;15:2823–2840. 34245122 10.1002/1878-0261.13056 PMC8564642

[8] Meads MB , Gatenby RA , Dalton WS . Environment‐mediated drug resistance: a major contributor to minimal residual disease. Nat Rev Cancer. 2009;9:665–674. 19693095 10.1038/nrc2714

[9] Vitale I , Pietrocola F , Guilbaud E , Aaronson SA , Abrams JM , Adam D , et al. Apoptotic cell death in disease‐current understanding of the NCCD 2023. Cell Death Differ. 2023;30:1097–1154. 37100955 10.1038/s41418-023-01153-w PMC10130819

[10] Toole BP . Hyaluronan: from extracellular glue to pericellular cue. Nat Rev Cancer. 2004;4:528–539. 15229478 10.1038/nrc1391

[11] Tian X , Azpurua J , Hine C , Vaidya A , Myakishev‐Rempel M , Ablaeva J , et al. High‐molecular‐mass hyaluronan mediates the cancer resistance of the naked mole rat. Nature. 2013;499:346–349. 23783513 10.1038/nature12234 PMC3720720

[12] Price ZK , Lokman NA , Ricciardelli C . Differing roles of Hyaluronan molecular weight on cancer cell behavior and chemotherapy resistance. Cancers (Basel). 2018;10:482. 30513961 10.3390/cancers10120482 PMC6316154

[13] Meyer K , Palmer JW . The polysaccharide of the vitreous humor. J Biol Chem. 1934;107:629–634.

[14] Engstrom‐Laurent A . Hyaluronan in joint disease. J Intern Med. 1997;242:57–60. 9260567 10.1046/j.1365-2796.1997.00174.x

[15] Tammi R , Agren UM , Tuhkanen AL , Tammi M . Hyaluronan metabolism in skin. Prog Histochem Cytochem. 1994;29:1–81. 10.1016/s0079-6336(11)80023-9 7892506

[16] Cowman MK , Lee HG , Schwertfeger KL , McCarthy JB , Turley EA . The content and size of Hyaluronan in biological fluids and tissues. Front Immunol. 2015;6:261. 26082778 10.3389/fimmu.2015.00261 PMC4451640

[17] Yuan H , Amin R , Ye X , de la Motte CA , Cowman MK . Determination of hyaluronan molecular mass distribution in human breast milk. Anal Biochem. 2015;474:78–88. 25579786 10.1016/j.ab.2014.12.020 PMC4357551

[18] Aya KL , Stern R . Hyaluronan in wound healing: rediscovering a major player. Wound Repair Regen. 2014;22:579–593. 25039417 10.1111/wrr.12214

[19] Frenkel JS . The role of hyaluronan in wound healing. Int Wound J. 2014;11:159–163. 22891615 10.1111/j.1742-481X.2012.01057.x PMC7950635

[20] Bohaumilitzky L , Huber AK , Stork EM , Wengert S , Woelfl F , Boehm H . A trickster in disguise: Hyaluronan's ambivalent roles in the matrix. Front Oncol. 2017;7:242. 29062810 10.3389/fonc.2017.00242 PMC5640889

[21] Karamanos NK , Piperigkou Z , Theocharis AD , Watanabe H , Franchi M , Baud S , et al. Proteoglycan chemical diversity drives multifunctional cell regulation and therapeutics. Chem Rev. 2018;118:9152–9232. 30204432 10.1021/acs.chemrev.8b00354

[22] Stern R , Asari AA , Sugahara KN . Hyaluronan fragments: an information‐rich system. Eur J Cell Biol. 2006;85:699–715. 16822580 10.1016/j.ejcb.2006.05.009

[23] Tammi MI , Day AJ , Turley EA . Hyaluronan and homeostasis: a balancing act. J Biol Chem. 2002;277:4581–4584. 11717316 10.1074/jbc.R100037200

[24] Tavianatou AG , Caon I , Franchi M , Piperigkou Z , Galesso D , Karamanos NK . Hyaluronan: molecular size‐dependent signaling and biological functions in inflammation and cancer. FEBS J. 2019;286:2883–2908. 30724463 10.1111/febs.14777

[25] Yang C , Cao M , Liu H , He Y , Xu J , Du Y , et al. The high and low molecular weight forms of hyaluronan have distinct effects on CD44 clustering. J Biol Chem. 2012;287:43094–43107. 23118219 10.1074/jbc.M112.349209 PMC3522304

[26] Akmal M , Singh A , Anand A , Kesani A , Aslam N , Goodship A , et al. The effects of hyaluronic acid on articular chondrocytes. J Bone Joint Surg Br. 2005;87:1143–1149. 16049255 10.1302/0301-620X.87B8.15083

[27] Kothapalli D , Zhao L , Hawthorne EA , Cheng Y , Lee E , Pure E , et al. Hyaluronan and CD44 antagonize mitogen‐dependent cyclin D1 expression in mesenchymal cells. J Cell Biol. 2007;176:535–544. 17296798 10.1083/jcb.200611058 PMC2063987

[28] Zhao YF , Qiao SP , Shi SL , Yao LF , Hou XL , Li CF , et al. Modulating three‐dimensional microenvironment with Hyaluronan of different molecular weights alters breast cancer cell invasion behavior. ACS Appl Mater Interfaces. 2017;9:9327–9338. 28240531 10.1021/acsami.6b15187

[29] Rayahin JE , Buhrman JS , Zhang Y , Koh TJ , Gemeinhart RA . High and low molecular weight hyaluronic acid differentially influence macrophage activation. ACS Biomater Sci Eng. 2015;1:481–493. 26280020 10.1021/acsbiomaterials.5b00181 PMC4533115

[30] Weigel PH . Hyaluronan synthase: the mechanism of initiation at the reducing end and a pendulum model for Polysaccharide translocation to the cell exterior. Int J Cell Biol. 2015;2015:367579. 26472958 10.1155/2015/367579 PMC4581545

[31] Prehm P , Schumacher U . Inhibition of hyaluronan export from human fibroblasts by inhibitors of multidrug resistance transporters. Biochem Pharmacol. 2004;68:1401–1410. 15345330 10.1016/j.bcp.2004.06.017

[32] Schulz T , Schumacher U , Prehm P . Hyaluronan export by the ABC transporter MRP5 and its modulation by intracellular cGMP. J Biol Chem. 2007;282:20999–21004. 17540771 10.1074/jbc.M700915200

[33] Camenisch TD , Spicer AP , Brehm‐Gibson T , Biesterfeldt J , Augustine ML , Calabro A Jr , et al. Disruption of hyaluronan synthase‐2 abrogates normal cardiac morphogenesis and hyaluronan‐mediated transformation of epithelium to mesenchyme. J Clin Invest. 2000;106:349–360. 10930438 10.1172/JCI10272 PMC314332

[34] Bao X , Ran J , Kong C , Wan Z , Wang J , Yu T , et al. Pan‐cancer analysis reveals the potential of hyaluronate synthase as therapeutic targets in human tumors. Heliyon. 2023;9:e19112. 37636435 10.1016/j.heliyon.2023.e19112 PMC10448108

[35] Tien JY , Spicer AP . Three vertebrate hyaluronan synthases are expressed during mouse development in distinct spatial and temporal patterns. Dev Dyn. 2005;233:130–141. 15765504 10.1002/dvdy.20328

[36] Auvinen P , Rilla K , Tumelius R , Tammi M , Sironen R , Soini Y , et al. Hyaluronan synthases (HAS1‐3) in stromal and malignant cells correlate with breast cancer grade and predict patient survival. Breast Cancer Res Treat. 2014;143:277–286. 24337597 10.1007/s10549-013-2804-7

[37] Yamada Y , Itano N , Narimatsu H , Kudo T , Morozumi K , Hirohashi S , et al. Elevated transcript level of hyaluronan synthase1 gene correlates with poor prognosis of human colon cancer. Clin Exp Metastasis. 2004;21:57–63. 15065603 10.1023/b:clin.0000017203.71293.e0

[38] Tammi RH , Passi AG , Rilla K , Karousou E , Vigetti D , Makkonen K , et al. Transcriptional and post‐translational regulation of hyaluronan synthesis. FEBS J. 2011;278:1419–1428. 21362137 10.1111/j.1742-4658.2011.08070.x

[39] Zhang L , Ji QH , Ruge F , Lane C , Morris D , Tee AR , et al. Reversal of pathological features of Graves' Orbitopathy by activation of Forkhead transcription factors, FOXOs. J Clin Endocrinol Metab. 2016;101:113–121. 10.1210/jc.2015-2932 26502358

[40] Chen L , Neville RD , Michael DR , Martin J , Luo DD , Thomas DW , et al. Identification and analysis of the human hyaluronan synthase 1 gene promoter reveals Smad3‐and Sp3‐mediated transcriptional induction. Matrix Biol. 2012;31:373–379. 23123404 10.1016/j.matbio.2012.10.002

[41] Wang S , Zhen L , Liu Z , Ai Q , Ji Y , Du G , et al. Identification and analysis of the promoter region of the human HAS3 gene. Biochem Biophys Res Commun. 2015;460:1008–1014. 25843802 10.1016/j.bbrc.2015.03.142

[42] Gatti V , Bongiorno‐Borbone L , Fierro C , Annicchiarico‐Petruzzelli M , Melino G , Peschiaroli A . p63 at the crossroads between stemness and metastasis in breast cancer. Int J Mol Sci. 2019;20:2683. 31159154 10.3390/ijms20112683 PMC6600246

[43] Gatti V , Fierro C , Annicchiarico‐Petruzzelli M , Melino G , Peschiaroli A . DeltaNp63 in squamous cell carcinoma: defining the oncogenic routes affecting epigenetic landscape and tumour microenvironment. Mol Oncol. 2019;13:981–1001. 30845357 10.1002/1878-0261.12473 PMC6487733

[44] Gatti V , Fierro C , Compagnone M , La Banca V , Mauriello A , Montanaro M , et al. DeltaNp63‐Senataxin circuit controls keratinocyte differentiation by promoting the transcriptional termination of epidermal genes. Proc Natl Acad Sci USA. 2022;119:e2104718119. 35235452 10.1073/pnas.2104718119 PMC8915885

[45] Kennedy MC , Lowe SW . Mutant p53: it's not all one and the same. Cell Death Differ. 2022;29:983–987. 35361963 10.1038/s41418-022-00989-y PMC9090915

[46] Levine AJ . Exploring the future of research in the Tp53 field. Cell Death Differ. 2022;29:893–894. 35383291 10.1038/s41418-022-00986-1 PMC9090764

[47] Mammarella E , Zampieri C , Panatta E , Melino G , Amelio I . NUAK2 and RCan2 participate in the p53 mutant pro‐tumorigenic network. Biol Direct. 2021;16:11. 34348766 10.1186/s13062-021-00296-5 PMC8335924

[48] Rozenberg JM , Zvereva S , Dalina A , Blatov I , Zubarev I , Luppov D , et al. The p53 family member p73 in the regulation of cell stress response. Biol Direct. 2021;16:23. 34749806 10.1186/s13062-021-00307-5 PMC8577020

[49] Thomas AF , Kelly GL , Strasser A . Of the many cellular responses activated by TP53, which ones are critical for tumour suppression? Cell Death Differ. 2022;29:961–971. 35396345 10.1038/s41418-022-00996-z PMC9090748

[50] Compagnone M , Gatti V , Presutti D , Ruberti G , Fierro C , Markert EK , et al. Delta Np63‐mediated regulation of hyaluronic acid metabolism and signaling supports HNSCC tumorigenesis. Proc Natl Acad Sci USA. 2017;114:13254–13259. 29162693 10.1073/pnas.1711777114 PMC5740608

[51] Gatti V , Fierro C , Compagnone M , Giangrazi F , Markert EK , Bongiorno‐Borbone L , et al. DeltaNp63 regulates the expression of hyaluronic acid‐related genes in breast cancer cells. Oncogenesis. 2018;7:65. 30139970 10.1038/s41389-018-0073-3 PMC6107578

[52] Deen AJ , Arasu UT , Pasonen‐Seppanen S , Hassinen A , Takabe P , Wojciechowski S , et al. UDP‐sugar substrates of HAS3 regulate its O‐GlcNAcylation, intracellular traffic, extracellular shedding and correlate with melanoma progression. Cell Mol Life Sci. 2016;73:3183–3204. 26883802 10.1007/s00018-016-2158-5 PMC11108457

[53] Karousou E , Kamiryo M , Skandalis SS , Ruusala A , Asteriou T , Passi A , et al. The activity of hyaluronan synthase 2 is regulated by dimerization and ubiquitination. J Biol Chem. 2010;285:23647–23654. 20507985 10.1074/jbc.M110.127050 PMC2911313

[54] Mehic M , de Sa VK , Hebestreit S , Heldin CH , Heldin P . The deubiquitinating enzymes USP4 and USP17 target hyaluronan synthase 2 and differentially affect its function. Oncogenesis. 2017;6:e348. 28604766 10.1038/oncsis.2017.45 PMC5519194

[55] Vigetti D , Deleonibus S , Moretto P , Karousou E , Viola M , Bartolini B , et al. Role of UDP‐N‐acetylglucosamine (GlcNAc) and O‐GlcNAcylation of hyaluronan synthase 2 in the control of chondroitin sulfate and hyaluronan synthesis. J Biol Chem. 2012;287:35544–35555. 22887999 10.1074/jbc.M112.402347 PMC3471761

[56] Jokela T , Karna R , Rauhala L , Bart G , Pasonen‐Seppanen S , Oikari S , et al. Human keratinocytes respond to extracellular UTP by induction of Hyaluronan synthase 2 expression and increased Hyaluronan synthesis. J Biol Chem. 2017;292:4861–4872. 28188289 10.1074/jbc.M116.760322 PMC5377801

[57] Jokela TA , Karna R , Makkonen KM , Laitinen JT , Tammi RH , Tammi MI . Extracellular UDP‐glucose activates P2Y14 receptor and induces signal transducer and activator of transcription 3 (STAT3) Tyr705 phosphorylation and binding to hyaluronan synthase 2 (HAS2) promoter, stimulating hyaluronan synthesis of keratinocytes. J Biol Chem. 2014;289:18569–18581. 24847057 10.1074/jbc.M114.551804 PMC4140273

[58] Rauhala L , Jokela T , Karna R , Bart G , Takabe P , Oikari S , et al. Extracellular ATP activates hyaluronan synthase 2 (HAS2) in epidermal keratinocytes via P2Y(2), Ca(2+) signaling, and MAPK pathways. Biochem J. 2018;475:1755–1772. 29626161 10.1042/BCJ20180054

[59] Vigetti D , Clerici M , Deleonibus S , Karousou E , Viola M , Moretto P , et al. Hyaluronan synthesis is inhibited by adenosine monophosphate‐activated protein kinase through the regulation of HAS2 activity in human aortic smooth muscle cells. J Biol Chem. 2011;286:7917–7924. 21228273 10.1074/jbc.M110.193656 PMC3048678

[60] Stern R , Jedrzejas MJ . Hyaluronidases: their genomics, structures, and mechanisms of action. Chem Rev. 2006;106:818–839. 16522010 10.1021/cr050247k PMC2547145

[61] Yoshida H , Nagaoka A , Kusaka‐Kikushima A , Tobiishi M , Kawabata K , Sayo T , et al. KIAA1199, a deafness gene of unknown function, is a new hyaluronan binding protein involved in hyaluronan depolymerization. Proc Natl Acad Sci USA. 2013;110:5612–5617. 23509262 10.1073/pnas.1215432110 PMC3619336

[62] Stern R . Hyaluronidases in cancer biology. Semin Cancer Biol. 2008;18:275–280. 18485730 10.1016/j.semcancer.2008.03.017

[63] Cherr GN , Yudin AI , Overstreet JW . The dual functions of GPI‐anchored PH‐20: hyaluronidase and intracellular signaling. Matrix Biol. 2001;20:515–525. 11731269 10.1016/s0945-053x(01)00171-8

[64] Soltes L , Mendichi R , Kogan G , Schiller J , Stankovska M , Arnhold J . Degradative action of reactive oxygen species on hyaluronan. Biomacromolecules. 2006;7:659–668. 16529395 10.1021/bm050867v

[65] Ghatak S , Maytin EV , Mack JA , Hascall VC , Atanelishvili I , Moreno Rodriguez R , et al. Roles of proteoglycans and Glycosaminoglycans in wound healing and fibrosis. Int J Cell Biol. 2015;2015:834893. 26448760 10.1155/2015/834893 PMC4581578

[66] Kao JJ . The NF‐kappaB inhibitor pyrrolidine dithiocarbamate blocks IL‐1beta induced hyaluronan synthase 1 (HAS1) mRNA transcription, pointing at NF‐kappaB dependence of the gene HAS1. Exp Gerontol. 2006;41:641–647. 16723203 10.1016/j.exger.2006.04.003

[67] McAtee CO , Barycki JJ , Simpson MA . Emerging roles for hyaluronidase in cancer metastasis and therapy. Adv Cancer Res. 2014;123:1–34. 25081524 10.1016/B978-0-12-800092-2.00001-0 PMC4445717

[68] Ooki T , Murata‐Kamiya N , Takahashi‐Kanemitsu A , Wu W , Hatakeyama M . High‐molecular‐weight Hyaluronan is a Hippo pathway ligand directing cell density‐dependent growth inhibition via PAR1b. Dev Cell. 2019;49:590–604.e9. 31080060 10.1016/j.devcel.2019.04.018

[69] Infante JR , Korn RL , Rosen LS , LoRusso P , Dychter SS , Zhu J , et al. Phase 1 trials of PEGylated recombinant human hyaluronidase PH20 in patients with advanced solid tumours. Br J Cancer. 2018;118:153–161.e3. 28949957 10.1038/bjc.2017.327 PMC5785735

[70] Carvalho AM , Reis RL , Pashkuleva I . Hyaluronan receptors as mediators and modulators of the tumor microenvironment. Adv Healthc Mater. 2023;12:e2202118. 36373221 10.1002/adhm.202202118 PMC11469756

[71] Giraud J , Seeneevassen L , Rousseau B , Bouriez D , Sifré E , Giese A , et al. CD44v3 is a marker of invasive cancer stem cells driving metastasis in gastric carcinoma. Gastric Cancer. 2023;26:234–249. 36528833 10.1007/s10120-022-01357-y PMC9950191

[72] Min J , Zhang C , Bliton RJ , Caldwell B , Caplan L , Presentation KS , et al. Dysplastic stem cell plasticity functions as a driving force for neoplastic transformation of precancerous gastric mucosa. Gastroenterology. 2022;163:875–890. 35700772 10.1053/j.gastro.2022.06.021 PMC9509466

[73] Chanmee T , Ontong P , Kimata K , Itano N . Key roles of Hyaluronan and its CD44 receptor in the Stemness and survival of cancer stem cells. Front Oncol. 2015;5:180. 26322272 10.3389/fonc.2015.00180 PMC4530590

[74] Thorne RF , Legg JW , Isacke CM . The role of the CD44 transmembrane and cytoplasmic domains in co‐ordinating adhesive and signalling events. J Cell Sci. 2004;117:373–380. 14702383 10.1242/jcs.00954

[75] Song JM , Im J , Nho RS , Han YH , Upadhyaya P , Kassie F . Hyaluronan‐CD44/RHAMM interaction‐dependent cell proliferation and survival in lung cancer cells. Mol Carcinog. 2019;58:321–333. 30365189 10.1002/mc.22930 PMC11005861

[76] Chen L , Bourguignon LY . Hyaluronan‐CD44 interaction promotes c‐Jun signaling and miRNA21 expression leading to Bcl‐2 expression and chemoresistance in breast cancer cells. Mol Cancer. 2014;13:52. 24606718 10.1186/1476-4598-13-52 PMC3975292

[77] Bourguignon LY , Earle C , Wong G , Spevak CC , Krueger K . Stem cell marker (Nanog) and Stat‐3 signaling promote MicroRNA‐21 expression and chemoresistance in hyaluronan/CD44‐activated head and neck squamous cell carcinoma cells. Oncogene. 2012;31:149–160. 21685938 10.1038/onc.2011.222 PMC3179812

[78] Bourguignon LYW , Earle C , Shiina M . Hyaluronan‐CD44 interaction promotes HPV 16 E6 oncogene‐mediated oropharyngeal cell carcinoma survival and chemoresistance. Matrix Biol. 2019;78–79:180–200. 10.1016/j.matbio.2018.07.008 30077625

[79] Chen C , Zhao S , Karnad A , Freeman JW . The biology and role of CD44 in cancer progression: therapeutic implications. J Hematol Oncol. 2018;11:64. 29747682 10.1186/s13045-018-0605-5 PMC5946470

[80] Mateo F , He Z , Mei L , de Garibay GR , Herranz C , Garcia N , et al. Modification of BRCA1‐associated breast cancer risk by HMMR overexpression. Nat Commun. 2022;13:1895. 35393420 10.1038/s41467-022-29335-z PMC8989921

[81] Bagli DJ , Joyner BD , Mahoney SR , McCulloch L . The hyaluronic acid receptor RHAMM is induced by stretch injury of rat bladder in vivo and influences smooth muscle cell contraction in vitro [corrected]. J Urol. 1999;162:832–840. 10458391 10.1097/00005392-199909010-00071

[82] Carvalho AM , Soares da Costa D , Reis RL , Pashkuleva I . RHAMM expression tunes the response of breast cancer cell lines to hyaluronan. Acta Biomater. 2022;146:187–196. 35577044 10.1016/j.actbio.2022.05.013

[83] Zlobec I , Terracciano L , Tornillo L , Günthert U , Vuong T , Jass JR , et al. Role of RHAMM within the hierarchy of well‐established prognostic factors in colorectal cancer. Gut. 2008;57:1413–1419. 18436576 10.1136/gut.2007.141192

[84] Choi S , Wang D , Chen X , Tang LH , Verma A , Chen Z , et al. Function and clinical relevance of RHAMM isoforms in pancreatic tumor progression. Mol Cancer. 2019;18:92. 31072393 10.1186/s12943-019-1018-y PMC6506944

[85] Wang C , Thor AD , Moore DH 2nd , Zhao Y , Kerschmann R , Stern R , et al. The overexpression of RHAMM, a hyaluronan‐binding protein that regulates ras signaling, correlates with overexpression of mitogen‐activated protein kinase and is a significant parameter in breast cancer progression. Clin Cancer Res. 1998;4:567–576. 9533523

[86] Maxwell CA , Rasmussen E , Zhan F , Keats JJ , Adamia S , Strachan E , et al. RHAMM expression and isoform balance predict aggressive disease and poor survival in multiple myeloma. Blood. 2004;104:1151–1158. 15105292 10.1182/blood-2003-11-4079

[87] Rozengurt E , Sinnett‐Smith J , Eibl G . Yes‐associated protein (YAP) in pancreatic cancer: at the epicenter of a targetable signaling network associated with patient survival. Signal Transduct Target Ther. 2018;3:11. 29682330 10.1038/s41392-017-0005-2 PMC5908807

[88] Zhu SW , Wang S , Wu ZZ , Yang QC , Chen DR , Wan SC , et al. Overexpression of CD168 is related to poor prognosis in oral squamous cell carcinoma. Oral Dis. 2022;28:364–372. 33386685 10.1111/odi.13766

[89] Crainie M , Belch AR , Mant MJ , Pilarski LM . Overexpression of the receptor for hyaluronan‐mediated motility (RHAMM) characterizes the malignant clone in multiple myeloma: identification of three distinct RHAMM variants. Blood. 1999;93:1684–1696. 10029598

[90] Du YC , Chou CK , Klimstra DS , Varmus H . Receptor for hyaluronan‐mediated motility isoform B promotes liver metastasis in a mouse model of multistep tumorigenesis and a tail vein assay for metastasis. Proc Natl Acad Sci USA. 2011;108:16753–16758. 21940500 10.1073/pnas.1114022108 PMC3189086

[91] Hall CL , Collis LA , Bo AJ , Lange L , McNicol A , Gerrard JM , et al. Fibroblasts require protein kinase C activation to respond to hyaluronan with increased locomotion. Matrix Biol. 2001;20:183–192. 11420150 10.1016/s0945-053x(01)00133-0

[92] Zhang H , Ren L , Ding Y , Li F , Chen X , Ouyang Y , et al. Hyaluronan‐mediated motility receptor confers resistance to chemotherapy via TGFbeta/Smad2‐induced epithelial‐mesenchymal transition in gastric cancer. FASEB J. 2019;33:6365–6377. 30802150 10.1096/fj.201802186R

[93] Liu Y , Li L , Wang L , Lu L , Li Y , Huang G , et al. ‘Two‐faces’ of hyaluronan, a dynamic barometer of disease progression in tumor microenvironment. Discov Oncol. 2023;14:11. 36698043 10.1007/s12672-023-00618-1 PMC9877274

[94] Ji X , Lu Y , Tian H , Meng X , Wei M , Cho WC . Chemoresistance mechanisms of breast cancer and their countermeasures. Biomed Pharmacother. 2019;114:108800. 30921705 10.1016/j.biopha.2019.108800

[95] Sudhesh Dev S , Zainal Abidin SA , Farghadani R , Othman I , Naidu R . Receptor tyrosine kinases and their signaling pathways as therapeutic targets of curcumin in cancer. Front Pharmacol. 2021;12:772510. 34867402 10.3389/fphar.2021.772510 PMC8634471

[96] Liu S , Cheng C . Akt signaling is sustained by a CD44 splice isoform‐mediated positive feedback loop. Cancer Res. 2017;77:3791–3801. 28533273 10.1158/0008-5472.CAN-16-2545 PMC5589713

[97] Ghatak S , Misra S , Toole BP . Hyaluronan constitutively regulates ErbB2 phosphorylation and signaling complex formation in carcinoma cells. J Biol Chem. 2005;280:8875–8883. 15632176 10.1074/jbc.M410882200

[98] Misra S , Ghatak S , Toole BP . Regulation of MDR1 expression and drug resistance by a positive feedback loop involving hyaluronan, phosphoinositide 3‐kinase, and ErbB2. J Biol Chem. 2005;280:20310–20315. 15784621 10.1074/jbc.M500737200

[99] Misra S , Obeid LM , Hannun YA , Minamisawa S , Berger FG , Markwald RR , et al. Hyaluronan constitutively regulates activation of COX‐2‐mediated cell survival activity in intestinal epithelial and colon carcinoma cells. J Biol Chem. 2008;283:14335–14344. 18326857 10.1074/jbc.M703811200 PMC2386915

[100] Torre C , Wang SJ , Xia W , Bourguignon LY . Reduction of hyaluronan‐CD44‐mediated growth, migration, and cisplatin resistance in head and neck cancer due to inhibition of rho kinase and PI‐3 kinase signaling. Arch Otolaryngol Head Neck Surg. 2010;136:493–501. 20479382 10.1001/archoto.2010.25 PMC2874205

[101] Santarpia L , Lippman SM , El‐Naggar AK . Targeting the MAPK‐RAS‐RAF signaling pathway in cancer therapy. Expert Opin Ther Targets. 2012;16:103–119. 22239440 10.1517/14728222.2011.645805 PMC3457779

[102] Wang SJ , Bourguignon LY . Hyaluronan and the interaction between CD44 and epidermal growth factor receptor in oncogenic signaling and chemotherapy resistance in head and neck cancer. Arch Otolaryngol Head Neck Surg. 2006;132:771–778. 16847188 10.1001/archotol.132.7.771

[103] Hamilton SR , Fard SF , Paiwand FF , Tolg C , Veiseh M , Wang C , et al. The hyaluronan receptors CD44 and Rhamm (CD168) form complexes with ERK1,2 that sustain high basal motility in breast cancer cells. J Biol Chem. 2007;282:16667–16680. 17392272 10.1074/jbc.M702078200 PMC2949353

[104] Vega FM , Ridley AJ . Rho GTPases in cancer cell biology. FEBS Lett. 2008;582:2093–2101. 18460342 10.1016/j.febslet.2008.04.039

[105] Hanna S , El‐Sibai M . Signaling networks of rho GTPases in cell motility. Cell Signal. 2013;25:1955–1961. 23669310 10.1016/j.cellsig.2013.04.009

[106] Bourguignon LY . Hyaluronan‐mediated CD44 activation of RhoGTPase signaling and cytoskeleton function promotes tumor progression. Semin Cancer Biol. 2008;18:251–259. 18450475 10.1016/j.semcancer.2008.03.007 PMC2505114

[107] Bourguignon LY , Wong G , Shiina M . Up‐regulation of histone Methyltransferase, DOT1L, by matrix Hyaluronan promotes MicroRNA‐10 expression leading to tumor cell invasion and Chemoresistance in cancer stem cells from head and neck squamous cell carcinoma. J Biol Chem. 2016;291:10571–10585. 27002147 10.1074/jbc.M115.700021 PMC4865907

[108] Bourguignon LY , Spevak CC , Wong G , Xia W , Gilad E . Hyaluronan‐CD44 interaction with protein kinase C(epsilon) promotes oncogenic signaling by the stem cell marker Nanog and the production of microRNA‐21, leading to down‐regulation of the tumor suppressor protein PDCD4, anti‐apoptosis, and chemotherapy resistance in breast tumor cells. J Biol Chem. 2009;284:26533–26546. 19633292 10.1074/jbc.M109.027466 PMC2785342

[109] Bourguignon LY , Peyrollier K , Xia W , Gilad E . Hyaluronan‐CD44 interaction activates stem cell marker Nanog, Stat‐3‐mediated MDR1 gene expression, and ankyrin‐regulated multidrug efflux in breast and ovarian tumor cells. J Biol Chem. 2008;283:17635–17651. 18441325 10.1074/jbc.M800109200 PMC2427357

[110] Turley EA . HIPPO and Hyaluronan: partners in tumor resistance? Bioessays. 2020;42:e2000090. 32449182 10.1002/bies.202000090

[111] Fletcher JI , Williams RT , Henderson MJ , Norris MD , Haber M . ABC transporters as mediators of drug resistance and contributors to cancer cell biology. Drug Resist Updat. 2016;26:1–9. 27180306 10.1016/j.drup.2016.03.001

[112] Thomas H , Coley HM . Overcoming multidrug resistance in cancer: an update on the clinical strategy of inhibiting p‐glycoprotein. Cancer Control. 2003;10:159–165. 12712010 10.1177/107327480301000207

[113] Bourguignon LYW , Xia W , Wong G . Hyaluronan‐mediated CD44 interaction with p300 and SIRT1 regulates beta‐catenin signaling and NFkappaB‐specific transcription activity leading to MDR1 and Bcl‐xL gene expression and chemoresistance in breast tumor cells. J Biol Chem. 2009;284:2657–2671. 19047049 10.1074/jbc.M806708200 PMC2631959

[114] Cole SP , Bhardwaj G , Gerlach JH , Mackie JE , Grant CE , Almquist KC , et al. Overexpression of a transporter gene in a multidrug‐resistant human lung cancer cell line. Science. 1992;258:1650–1654. 1360704 10.1126/science.1360704

[115] Ohashi R , Takahashi F , Cui R , Yoshioka M , Gu T , Sasaki S , et al. Interaction between CD44 and hyaluronate induces chemoresistance in non‐small cell lung cancer cell. Cancer Lett. 2007;252:225–234. 17276588 10.1016/j.canlet.2006.12.025

[116] Ricciardelli C , Ween MP , Lokman NA , Tan IA , Pyragius CE , Oehler MK . Chemotherapy‐induced hyaluronan production: a novel chemoresistance mechanism in ovarian cancer. BMC Cancer. 2013;13:476. 24124770 10.1186/1471-2407-13-476 PMC3852938

[117] Gilg AG , Tye SL , Tolliver LB , Wheeler WG , Visconti RP , Duncan JD , et al. Targeting hyaluronan interactions in malignant gliomas and their drug‐resistant multipotent progenitors. Clin Cancer Res. 2008;14:1804–1813. 18347183 10.1158/1078-0432.CCR-07-1228

[118] Slomiany MG , Dai L , Bomar PA , Knackstedt TJ , Kranc DA , Tolliver L , et al. Abrogating drug resistance in malignant peripheral nerve sheath tumors by disrupting hyaluronan‐CD44 interactions with small hyaluronan oligosaccharides. Cancer Res. 2009;69:4992–4998. 19470767 10.1158/0008-5472.CAN-09-0143 PMC3655760

[119] Miletti‐Gonzalez KE , Chen S , Muthukumaran N , Saglimbeni GN , Wu X , Yang J , et al. The CD44 receptor interacts with P‐glycoprotein to promote cell migration and invasion in cancer. Cancer Res. 2005;65:6660–6667. 16061646 10.1158/0008-5472.CAN-04-3478

[120] Huang T , Song X , Xu D , Tiek D , Goenka A , Wu B , et al. Stem cell programs in cancer initiation, progression, and therapy resistance. Theranostics. 2020;10:8721–8743. 32754274 10.7150/thno.41648 PMC7392012

[121] Yang L , Shi P , Zhao G , Xu J , Peng W , Zhang J , et al. Targeting cancer stem cell pathways for cancer therapy. Signal Transduct Target Ther. 2020;5:8. 32296030 10.1038/s41392-020-0110-5 PMC7005297

[122] Shiina M , Bourguignon LY . Selective activation of cancer stem cells by size‐specific Hyaluronan in head and neck cancer. Int J Cell Biol. 2015;2015:989070. 26448762 10.1155/2015/989070 PMC4581563

[123] Bongiorno‐Borbone L , Giacobbe A , Compagnone M , Eramo A , De Maria R , Peschiaroli A , et al. Anti‐tumoral effect of desmethylclomipramine in lung cancer stem cells. Oncotarget. 2015;6:16926–16938. 26219257 10.18632/oncotarget.4700 PMC4627282

[124] Lendeckel U , Wolke C . Redox‐regulation in cancer stem cells. Biomedicine. 2022;10:2413. 10.3390/biomedicines10102413 PMC9598867 36289675

[125] Muller F , Lim JKM , Bebber CM , Seidel E , Tishina S , Dahlhaus A , et al. Elevated FSP1 protects KRAS‐mutated cells from ferroptosis during tumor initiation. Cell Death Differ. 2023;30:442–456. 36443441 10.1038/s41418-022-01096-8 PMC9950476

[126] Carnero A , Garcia‐Mayea Y , Mir C , Lorente J , Rubio IT , LLeonart ME . The cancer stem‐cell signaling network and resistance to therapy. Cancer Treat Rev. 2016;49:25–36. 27434881 10.1016/j.ctrv.2016.07.001

[127] Arena A , Romeo MA , Benedetti R , Gilardini Montani MS , Santarelli R , Gonnella R , et al. NRF2 and STAT3: friends or foes in carcinogenesis? Discov Oncol. 2023;14:37. 37000324 10.1007/s12672-023-00644-z PMC10064365

[128] Awuah WA , Toufik AR , Yarlagadda R , Mikhailova T , Mehta A , Huang H , et al. Exploring the role of Nrf2 signaling in glioblastoma multiforme. Discov Oncol. 2022;13:94. 36169772 10.1007/s12672-022-00556-4 PMC9519816

[129] Xu L , Zhu Y , Li C , Wang Q , Ma L , Wang J , et al. Small extracellular vesicles derived from Nrf2‐overexpressing human amniotic mesenchymal stem cells protect against lipopolysaccharide‐induced acute lung injury by inhibiting NLRP3. Biol Direct. 2022;17:35. 36447296 10.1186/s13062-022-00351-9 PMC9706911

[130] Ryoo IG , Choi BH , Ku SK , Kwak MK . High CD44 expression mediates p62‐associated NFE2L2/NRF2 activation in breast cancer stem cell‐like cells: implications for cancer stem cell resistance. Redox Biol. 2018;17:246–258. 29729523 10.1016/j.redox.2018.04.015 PMC6006726

[131] Ishimoto T , Nagano O , Yae T , Tamada M , Motohara T , Oshima H , et al. CD44 variant regulates redox status in cancer cells by stabilizing the xCT subunit of system xc(−) and thereby promotes tumor growth. Cancer Cell. 2011;19:387–400. 21397861 10.1016/j.ccr.2011.01.038

[132] Hagiwara M , Kikuchi E , Tanaka N , Kosaka T , Mikami S , Saya H , et al. Variant isoforms of CD44 involves acquisition of chemoresistance to cisplatin and has potential as a novel indicator for identifying a cisplatin‐resistant population in urothelial cancer. BMC Cancer. 2018;18:113. 29385995 10.1186/s12885-018-3988-3 PMC5793458

[133] Hasegawa M , Takahashi H , Rajabi H , Alam M , Suzuki Y , Yin L , et al. Functional interactions of the cystine/glutamate antiporter, CD44v and MUC1‐C oncoprotein in triple‐negative breast cancer cells. Oncotarget. 2016;7:11756–11769. 26930718 10.18632/oncotarget.7598 PMC4914246

[134] Skandalis SS , Kozlova I , Engstrom U , Hellman U , Heldin P . Proteomic identification of CD44 interacting proteins. IUBMB Life. 2010;62:833–840. 21117172 10.1002/iub.392

[135] Tamada M , Nagano O , Tateyama S , Ohmura M , Yae T , Ishimoto T , et al. Modulation of glucose metabolism by CD44 contributes to antioxidant status and drug resistance in cancer cells. Cancer Res. 2012;72:1438–1448. 22293754 10.1158/0008-5472.CAN-11-3024

[136] Provenzano PP , Cuevas C , Chang AE , Goel VK , Von Hoff DD , Hingorani SR . Enzymatic targeting of the stroma ablates physical barriers to treatment of pancreatic ductal adenocarcinoma. Cancer Cell. 2012;21:418–429. 22439937 10.1016/j.ccr.2012.01.007 PMC3371414

[137] Jacobetz MA , Chan DS , Neesse A , Bapiro TE , Cook N , Frese KK , et al. Hyaluronan impairs vascular function and drug delivery in a mouse model of pancreatic cancer. Gut. 2013;62:112–120. 22466618 10.1136/gutjnl-2012-302529 PMC3551211

[138] Thompson CB , Shepard HM , O'Connor PM , Kadhim S , Jiang P , Osgood RJ , et al. Enzymatic depletion of tumor hyaluronan induces antitumor responses in preclinical animal models. Mol Cancer Ther. 2010;9:3052–3064. 20978165 10.1158/1535-7163.MCT-10-0470

[139] Liu Z , Hou P , Fang J , Zhu J , Zha J , Liu R , et al. Mesenchymal stromal cells confer breast cancer doxorubicin resistance by producing hyaluronan. Oncogene. 2023;42:3221–3235. 37704784 10.1038/s41388-023-02837-w

[140] Chen J , Meng J , Jin C , Mo F , Ding Y , Gao X , et al. 4‐Methylumbelliferone treatment and hyaluronan inhibition as a therapeutic strategy for chronic prostatitis. Prostate. 2021;81:1078–1090. 34320251 10.1002/pros.24205

[141] Kakizaki I , Kojima K , Takagaki K , Endo M , Kannagi R , Ito M , et al. A novel mechanism for the inhibition of hyaluronan biosynthesis by 4‐methylumbelliferone. J Biol Chem. 2004;279:33281–33289. 15190064 10.1074/jbc.M405918200

[142] Kultti A , Pasonen‐Seppanen S , Jauhiainen M , Rilla KJ , Karna R , Pyoria E , et al. 4‐Methylumbelliferone inhibits hyaluronan synthesis by depletion of cellular UDP‐glucuronic acid and downregulation of hyaluronan synthase 2 and 3. Exp Cell Res. 2009;315:1914–1923. 19285976 10.1016/j.yexcr.2009.03.002

[143] Arai E , Nishida Y , Wasa J , Urakawa H , Zhuo L , Kimata K , et al. Inhibition of hyaluronan retention by 4‐methylumbelliferone suppresses osteosarcoma cells in vitro and lung metastasis in vivo. Br J Cancer. 2011;105:1839–1849. 22045192 10.1038/bjc.2011.459 PMC3251882

[144] Karalis TT , Heldin P , Vynios DH , Neill T , Buraschi S , Iozzo RV , et al. Tumor‐suppressive functions of 4‐MU on breast cancer cells of different ER status: regulation of hyaluronan/HAS2/CD44 and specific matrix effectors. Matrix Biol. 2019;78–79:118–138. 10.1016/j.matbio.2018.04.007 29673760

[145] Lokeshwar VB , Lopez LE , Munoz D , Chi A , Shirodkar SP , Lokeshwar SD , et al. Antitumor activity of hyaluronic acid synthesis inhibitor 4‐methylumbelliferone in prostate cancer cells. Cancer Res. 2010;70:2613–2623. 20332231 10.1158/0008-5472.CAN-09-3185 PMC2848908

[146] Pibuel MA , Diaz M , Molinari Y , Poodts D , Silvestroff L , Lompardia SL , et al. 4‐Methylumbelliferone as a potent and selective antitumor drug on a glioblastoma model. Glycobiology. 2021;31:29–43. 32472122 10.1093/glycob/cwaa046

[147] Twarock S , Freudenberger T , Poscher E , Dai G , Jannasch K , Dullin C , et al. Inhibition of oesophageal squamous cell carcinoma progression by in vivo targeting of hyaluronan synthesis. Mol Cancer. 2011;10:30. 21429221 10.1186/1476-4598-10-30 PMC3078897

[148] Twarock S , Reichert C , Bach K , Reiners O , Kretschmer I , Gorski DJ , et al. Inhibition of the hyaluronan matrix enhances metabolic anticancer therapy by dichloroacetate in vitro and in vivo. Br J Pharmacol. 2019;176:4474–4490. 31351004 10.1111/bph.14808 PMC6932941

[149] Yates TJ , Lopez LE , Lokeshwar SD , Ortiz N , Kallifatidis G , Jordan A , et al. Dietary supplement 4‐methylumbelliferone: an effective chemopreventive and therapeutic agent for prostate cancer. J Natl Cancer Inst. 2015;107:djv085. 25868577 10.1093/jnci/djv085 PMC4554252

[150] Ricciardelli C , Lokman NA , Sabit I , Gunasegaran K , Bonner WM , Pyragius CE , et al. Novel ex vivo ovarian cancer tissue explant assay for prediction of chemosensitivity and response to novel therapeutics. Cancer Lett. 2018;421:51–58. 29425684 10.1016/j.canlet.2018.02.006

[151] Cho H , Matsumoto S , Fujita Y , Kuroda A , Menju T , Sonobe M , et al. Trametinib plus 4‐Methylumbelliferone exhibits antitumor effects by ERK blockade and CD44 downregulation and affects PD‐1 and PD‐L1 in malignant pleural mesothelioma. J Thorac Oncol. 2017;12:477–490. 27867002 10.1016/j.jtho.2016.10.023

[152] Malvicini M , Fiore E , Ghiaccio V , Piccioni F , Rizzo M , Olmedo Bonadeo L , et al. Tumor microenvironment remodeling by 4‐Methylumbelliferone boosts the antitumor effect of combined immunotherapy in murine colorectal carcinoma. Mol Ther. 2015;23:1444–1455. 26105158 10.1038/mt.2015.112 PMC4817882

[153] Abate A , Dimartino V , Spina P , Costa PL , Lombardo C , Santini A , et al. Hymecromone in the treatment of motor disorders of the bile ducts: a multicenter, double‐blind, placebo‐controlled clinical study. Drugs Exp Clin Res. 2001;27:223–231. 11951580

[154] Hoffmann RM , Schwarz G , Pohl C , Ziegenhagen DJ , Kruis W . Bile acid‐independent effect of hymecromone on bile secretion and common bile duct motility. Dtsch Med Wochenschr. 2005;130:1938–1943. 16123896 10.1055/s-2005-872606

[155] Diaz M , Pibuel M , Paglilla N , Poodts D , Alvarez E , Papademetrio DL , et al. 4‐Methylumbelliferone induces antitumor effects independently of hyaluronan synthesis inhibition in human acute leukemia cell lines. Life Sci. 2021;287:120065. 34678263 10.1016/j.lfs.2021.120065

[156] Tamura R , Yokoyama Y , Yoshida H , Imaizumi T , Mizunuma H . 4‐Methylumbelliferone inhibits ovarian cancer growth by suppressing thymidine phosphorylase expression. J Ovarian Res. 2014;7:94. 25304388 10.1186/s13048-014-0094-2 PMC4198731

[157] Rilla K , Pasonen‐Seppanen S , Rieppo J , Tammi M , Tammi R . The hyaluronan synthesis inhibitor 4‐methylumbelliferone prevents keratinocyte activation and epidermal hyperproliferation induced by epidermal growth factor. J Invest Dermatol. 2004;123:708–714. 15373776 10.1111/j.0022-202X.2004.23409.x

[158] Yoshioka Y , Kozawa E , Urakawa H , Arai E , Futamura N , Zhuo L , et al. Inhibition of hyaluronan synthesis alters sulfated glycosaminoglycans deposition during chondrogenic differentiation in ATDC5 cells. Histochem Cell Biol. 2015;144:167–177. 25929745 10.1007/s00418-015-1325-3

[159] Andreichenko IN , Tsitrina AA , Fokin AV , Gabdulkhakova AI , Maltsev DI , Perelman GS , et al. 4‐methylumbelliferone prevents liver fibrosis by affecting Hyaluronan deposition, FSTL1 expression and cell localization. Int J Mol Sci. 2019;20:6301. 31847129 10.3390/ijms20246301 PMC6941058

[160] Idota M , Ishizuka S , Hiraiwa H , Yamashita S , Oba H , Kawamura Y , et al. 4‐Methylumbelliferone suppresses catabolic activation in anterior cruciate ligament‐derived cells via a mechanism independent of hyaluronan inhibition. J Orthop Surg Res. 2021;16:507. 34404442 10.1186/s13018-021-02637-6 PMC8369759

[161] Vigetti D , Rizzi M , Viola M , Karousou E , Genasetti A , Clerici M , et al. The effects of 4‐methylumbelliferone on hyaluronan synthesis, MMP2 activity, proliferation, and motility of human aortic smooth muscle cells. Glycobiology. 2009;19:537–546. 19240269 10.1093/glycob/cwp022

[162] Nakamura T , Ishikawa T , Nanashima N , Miura T , Nozaka H , Nakaoka R , et al. 4‐Methylumbelliferone induces the expression of membrane type 1‐matrix metalloproteinase in cultured human skin fibroblasts. Biochem Biophys Res Commun. 2002;298:646–650. 12419303 10.1016/s0006-291x(02)02516-0

[163] Nakamura R , Kuwabara H , Yoneda M , Yoshihara S , Ishikawa T , Miura T , et al. Suppression of matrix metalloproteinase‐9 by 4‐methylumbelliferone. Cell Biol Int. 2007;31:1022–1026. 17470403 10.1016/j.cellbi.2007.03.016

[164] Barnes HW , Demirdjian S , Haddock NL , Kaber G , Martinez HA , Nagy N , et al. Hyaluronan in the pathogenesis of acute and post‐acute COVID‐19 infection. Matrix Biol. 2023;116:49–66. 36750167 10.1016/j.matbio.2023.02.001 PMC9899355

[165] Shi Y , Wang Y , Shao C , Huang J , Gan J , Huang X , et al. COVID‐19 infection: the perspectives on immune responses. Cell Death Differ. 2020;27:1451–1454. 32205856 10.1038/s41418-020-0530-3 PMC7091918

[166] Yang S , Ling Y , Zhao F , Li W , Song Z , Wang L , et al. Hymecromone: a clinical prescription hyaluronan inhibitor for efficiently blocking COVID‐19 progression. Signal Transduct Target Ther. 2022;7:91. 35304437 10.1038/s41392-022-00952-w PMC8931182

[167] Baumgartner G , Gomar‐Hoss C , Sakr L , Ulsperger E , Wogritsch C . The impact of extracellular matrix on the chemoresistance of solid tumors – experimental and clinical results of hyaluronidase as additive to cytostatic chemotherapy. Cancer Lett. 1998;131:85–99. 9839623

[168] Hingorani SR , Harris WP , Beck JT , Berdov BA , Wagner SA , Pshevlotsky EM , et al. Phase Ib study of PEGylated recombinant human hyaluronidase and gemcitabine in patients with advanced pancreatic cancer. Clin Cancer Res. 2016;22:2848–2854. 26813359 10.1158/1078-0432.CCR-15-2010 PMC7787348

[169] Hingorani SR , Zheng L , Bullock AJ , Seery TE , Harris WP , Sigal DS , et al. HALO 202: randomized phase II study of PEGPH20 plus nab‐paclitaxel/gemcitabine versus nab‐paclitaxel/gemcitabine in patients with untreated, metastatic pancreatic ductal adenocarcinoma. J Clin Oncol. 2018;36:359–366. 29232172 10.1200/JCO.2017.74.9564

[170] Klocker J , Sabitzer H , Raunik W , Wieser S , Schumer J . Hyaluronidase as additive to induction chemotherapy in advanced squamous cell carcinoma of the head and neck. Cancer Lett. 1998;131:113–115. 9839626 10.1016/s0304-3835(98)00207-9

[171] Ko AH , Kim KP , Siveke JT , Lopez CD , Lacy J , O'Reilly EM , et al. Atezolizumab plus PEGPH20 versus chemotherapy in advanced pancreatic ductal adenocarcinoma and gastric cancer: MORPHEUS phase Ib/II umbrella randomized study platform. Oncologist. 2023;28:553‐e472. 36940261 10.1093/oncolo/oyad022 PMC10243783

[172] Pillwein K , Fuiko R , Slavc I , Czech T , Hawliczek G , Bernhardt G , et al. Hyaluronidase additional to standard chemotherapy improves outcome for children with malignant brain tumors. Cancer Lett. 1998;131:101–108. 9839624 10.1016/s0304-3835(98)00205-5

[173] Smith KJ , Skelton HG , Turiansky G , Wagner KF . Hyaluronidase enhances the therapeutic effect of vinblastine in intralesional treatment of Kaposi's sarcoma. Military medical consortium for the advancement of retroviral research (MMCARR). J Am Acad Dermatol. 1997;36:239–242. 9039176 10.1016/s0190-9622(97)70288-3

[174] Van Cutsem E , Tempero MA , Sigal D , Oh DY , Fazio N , Macarulla T , et al. Randomized phase III trial of Pegvorhyaluronidase Alfa with nab‐paclitaxel plus gemcitabine for patients with Hyaluronan‐high metastatic pancreatic adenocarcinoma. J Clin Oncol. 2020;38:3185–3194. 32706635 10.1200/JCO.20.00590 PMC7499614

[175] Doherty GJ , Tempero M , Corrie PG . HALO‐109‐301: a phase III trial of PEGPH20 (with gemcitabine and nab‐paclitaxel) in hyaluronic acid‐high stage IV pancreatic cancer. Future Oncol. 2018;14:13–22. 10.2217/fon-2017-0338 29235360

[176] S D, Halozyme Announces HALO‐301 Phase 3 Study Fails to Meet Primary Endpoint. Halozyme Therapeutics, Web site. https://wwwhalozymecom/investors/news‐releases/news‐release‐details/2019/Halozyme‐Announces‐HALO‐301‐Phase‐3‐Study‐Fails‐To‐Meet‐Primary‐Endpoint/defaultaspx

[177] Ramanathan RK , McDonough SL , Philip PA , Hingorani SR , Lacy J , Kortmansky JS , et al. Phase IB/II randomized study of FOLFIRINOX plus Pegylated recombinant human hyaluronidase versus FOLFIRINOX alone in patients with metastatic pancreatic adenocarcinoma: SWOG S1313. J Clin Oncol. 2019;37:1062–1069. 30817250 10.1200/JCO.18.01295 PMC6494359

[178] Rhim AD , Oberstein PE , Thomas DH , Mirek ET , Palermo CF , Sastra SA , et al. Stromal elements act to restrain, rather than support, pancreatic ductal adenocarcinoma. Cancer Cell. 2014;25:735–747. 24856585 10.1016/j.ccr.2014.04.021 PMC4096698

[179] D'Arena G , Calapai G , Deaglio S . Anti‐CD44 mAb for the treatment of B‐cell chronic lymphocytic leukemia and other hematological malignancies: evaluation of WO2013063498. Expert Opin Ther Pat. 2014;24:821–828. 24798704 10.1517/13543776.2014.915942

[180] Khan F , Gurung S , Gunassekaran GR , Vadevoo SMP , Chi L , Permpoon U , et al. Identification of novel CD44v6‐binding peptides that block CD44v6 and deliver a pro‐apoptotic peptide to tumors to inhibit tumor growth and metastasis in mice. Theranostics. 2021;11:1326–1344. 33391537 10.7150/thno.50564 PMC7738880

[181] Shah V , Taratula O , Garbuzenko OB , Taratula OR , Rodriguez‐Rodriguez L , Minko T . Targeted nanomedicine for suppression of CD44 and simultaneous cell death induction in ovarian cancer: an optimal delivery of siRNA and anticancer drug. Clin Cancer Res. 2013;19:6193–6204. 24036854 10.1158/1078-0432.CCR-13-1536 PMC3846837

[182] Wallach‐Dayan SB , Rubinstein AM , Hand C , Breuer R , Naor D . DNA vaccination with CD44 variant isoform reduces mammary tumor local growth and lung metastasis. Mol Cancer Ther. 2008;7:1615–1623. 18566232 10.1158/1535-7163.MCT-07-2383

[183] Vahidian F , Safarzadeh E , Mohammadi A , Najjary S , Mansoori B , Majidi J , et al. siRNA‐mediated silencing of CD44 delivered by jet Pei enhanced doxorubicin chemo sensitivity and altered miRNA expression in human breast cancer cell line (MDA‐MB468). Mol Biol Rep. 2020;47:9541–9551. 33206362 10.1007/s11033-020-05952-z

[184] Riechelmann H , Sauter A , Golze W , Hanft G , Schroen C , Hoermann K , et al. Phase I trial with the CD44v6‐targeting immunoconjugate bivatuzumab mertansine in head and neck squamous cell carcinoma. Oral Oncol. 2008;44:823–829. 18203652 10.1016/j.oraloncology.2007.10.009

[185] Ahrens T , Sleeman JP , Schempp CM , Howells N , Hofmann M , Ponta H , et al. Soluble CD44 inhibits melanoma tumor growth by blocking cell surface CD44 binding to hyaluronic acid. Oncogene. 2001;20:3399–3408. 11423990 10.1038/sj.onc.1204435

[186] Zeng C , Toole BP , Kinney SD , Kuo JW , Stamenkovic I . Inhibition of tumor growth in vivo by hyaluronan oligomers. Int J Cancer. 1998;77:396–401. 9663602 10.1002/(sici)1097-0215(19980729)77:3<396::aid-ijc15>3.0.co;2-6

[187] Fuchs K , Hippe A , Schmaus A , Homey B , Sleeman JP , Orian‐Rousseau V . Opposing effects of high‐ and low‐molecular weight hyaluronan on CXCL12‐induced CXCR4 signaling depend on CD44. Cell Death Dis. 2013;4:e819. 24091662 10.1038/cddis.2013.364 PMC3824673

[188] Ghatak S , Misra S , Toole BP . Hyaluronan oligosaccharides inhibit anchorage‐independent growth of tumor cells by suppressing the phosphoinositide 3‐kinase/Akt cell survival pathway. J Biol Chem. 2002;277:38013–38020. 12145277 10.1074/jbc.M202404200

[189] Ashrafizadeh M , Mirzaei S , Gholami MH , Hashemi F , Zabolian A , Raei M , et al. Hyaluronic acid‐based nanoplatforms for doxorubicin: a review of stimuli‐responsive carriers, co‐delivery and resistance suppression. Carbohydr Polym. 2021;272:118491. 34420747 10.1016/j.carbpol.2021.118491

[190] Ossipov DA . Nanostructured hyaluronic acid‐based materials for active delivery to cancer. Expert Opin Drug Deliv. 2010;7:681–703. 20367530 10.1517/17425241003730399

[191] Gibbs P , Brown TJ , Ng R , Jennens R , Cinc E , Pho M , et al. A pilot human evaluation of a formulation of irinotecan and hyaluronic acid in 5‐fluorouracil‐refractory metastatic colorectal cancer patients. Chemotherapy. 2009;55:49–59. 19060478 10.1159/000180339

[192] Gibbs P , Clingan PR , Ganju V , Strickland AH , Wong SS , Tebbutt NC , et al. Hyaluronan‐Irinotecan improves progression‐free survival in 5‐fluorouracil refractory patients with metastatic colorectal cancer: a randomized phase II trial. Cancer Chemother Pharmacol. 2011;67:153–163. 20333384 10.1007/s00280-010-1303-3

[193] A B, Alchemia Announces Phase III Trial Results for HA‐Irinotecan in Metastatic Colorectal Cancer. Available from http://www.marketwired.com/press‐release/alchemia‐announces‐phase‐iii‐trial‐results‐ha‐irinotecan‐metastatic‐colorectal‐cancer‐asx‐acl‐1961090htm.

[194] Candeil L , Gourdier I , Peyron D , Vezzio N , Copois V , Bibeau F , et al. ABCG2 overexpression in colon cancer cells resistant to SN38 and in irinotecan‐treated metastases. Int J Cancer. 2004;109:848–854. 15027118 10.1002/ijc.20032

[195] Sahin IH , Klostergaard J . CD44 as a drug delivery target in human cancers: where are we now? Expert Opin Ther Targets. 2015;19:1587–1591. 26374284 10.1517/14728222.2015.1088834

